# Can imaging be the new yardstick for diagnosing peripheral neuropathy?—a comparison between high resolution ultrasound and MR neurography with an approach to diagnosis

**DOI:** 10.1186/s13244-019-0787-6

**Published:** 2019-11-01

**Authors:** Aakanksha Agarwal, Abhishek Chandra, Usha Jaipal, Meenu Bagarhatta, Kuldeep Mendiratta, Alka Goyal, Raghav Kumar, Naresh Mangalhara

**Affiliations:** 1 Department of Radiodiagnosis, SMS Medical College, Jaipur, India; 2 Department of Orthopaedics, SMS Medical College, Jaipur, India; 3 Malviya Nagar, Jaipur, India

**Keywords:** Peripheral nerve, Ultrasonography, Magnetic resonance imaging

## Abstract

**Purpose:**

Peripheral neuropathies are a group of disorders which affect the peripheral nervous system which have been conventionally diagnosed using electrodiagnostic studies. This study was carried out to assess the role of imaging in diagnosing peripheral mononeuropathy as exact anatomical localisation of the pathology is possible using high-resolution ultrasound and MR neurography, the modalities assessed in this study.

**Method:**

A hospital-based prospective analytical study was carried out in a resource-limited setting on 180 peripheral nerves in 131 patients with symptoms of peripheral mononeuropathy after taking IRB approval. Each patient underwent high-resolution ultrasound examination and MR neurography, findings of which were then compared and statistically analysed assuming electrodiagnostic findings as the gold standard.

**Results:**

Overall, the diagnostic accuracy was highest for the proton density fat-saturated MR sequence (93.89%) followed by high-resolution ultrasound (80%). The sensitivity was highest for proton density fat-saturated sequence while the T1 MR sequence had the highest specificity. Combined diagnostic accuracy of both modalities was calculated to be 93.33% with a negative predictive value of 80%. High-resolution ultrasound and MRI equally detected the cases with nerve discontinuity, while neuromas were better identified on MRI.

**Conclusion:**

With the advent of higher frequency probes and improved MR field strength, imaging of peripheral nerves is possible with better accuracy. Imaging assessment of nerves allows anatomical delineation with identification of exact site of involvement. This comparative study demonstrates the role of imaging in diagnosing peripheral nerve pathologies with the accuracy of MRI as high as 93.89% which may serve as an imaging gold standard. High-resolution ultrasound, being quicker, cost effective and a comparable accuracy of 80% can serve as a reliable screening tool. This study incorporates a larger study group and compares HRUS with MRI, taking NCV as gold standard, which has not been done in the preceding studies. With this study, we conclude that these two imaging modalities are not mutually exclusive. Rather, they complement each other and can be used in conjunction as an imaging yardstick for diagnosing peripheral neuropathies.

**Electronic supplementary material:**

The online version of this article (10.1186/s13244-019-0787-6) contains supplementary material, which is available to authorized users.

## Key points

- Advancement in imaging modalities and technology will usher in an era of multimodality approach to diagnosis of peripheral mononeuropathy with imaging providing the accurate anatomical details of the site of pathology.
- High-resolution ultrasound and MR neurography provide exact anatomical details about the affected nerve to aid in clinical management and decision making.
- These two modalities complement each other with MRI providing better soft tissue details and HRUS being readily available, quicker and cheaper.
- Traditionally, clinical examination and electrodiagnostic studies have been the mainstay of diagnosis. Imaging provides unparalleled complementary details which supplement the diagnosis.

## Introduction

Peripheral neuropathy is a disorder of nerve(s) apart from the brain and spinal cord which indicates any disorder of the peripheral nervous system with variable presentation and numerous causes. The usual presenting complaints of patients with peripheral neuropathy include tingling, unusual sensations, numbness, weakness or burning pain in the affected area. Mononeuropathy refers to a single peripheral nerve involvement and usually occurs due to trauma, compression or entrapment [1]. Any damage or disease affecting peripheral nerves in roughly the same areas on both sides of the body, manifesting as weakness, numbness and burning pain is referred to as polyneuropathy [1]. With the increase in rate of obesity and prevalence of type 2 diabetes, diabetic neuropathy has become the most common cause of peripheral neuropathy in developed countries [2].

Conventionally, the diagnosis of neuropathies is suspected clinically, monitored and confirmed electro-diagnostically [3]. An important clinical sign of assessment of nerve injury and degree of improvement is the Tinel’s sign. Seddon (1943) and Sunderland (1951) classified nerve injuries into five types to which Mackinnon and Dellon added a sixth type in 1992. This grade VI injury is as a mixed type of injury which denotes various types of injuries across the cross section of the nerve [4–6]. The different types of injuries are illustrated in Table 1 along with their imaging findings [7].

**Table 1:** Classification of nerve injury according to Seddon and Sunderland with corresponding findings on HRUS and MRI [10]

Seddon	Sunderland	Description	MRI	Ultrasound
Neuropraxia	I	Conduction block	T2 hyperintensity	Decreased echogenicity of nerve (hypoechoic)
Axonotmesis	II	Discontinuity of axon with Wallerian degeneration	T2 hyperintensity with increased size. Hyperintensity in muscles due to denervation.	Decreased echogenicity and increased calibre of the nerve
	III	Scarring of the endoneurium	Endoneurium can-not be delineated with current MR technique. T2 hyperintensity with increased size. Hyperintensity in muscles due to denervation.	Focal decrease in echogenicity with increase in calibre with change in echotexture of the affected muscles.
	IV	Neuroma in continuity with formation of scar which blocks nerve regeneration	T1 hypointense, T2 hyperintense focal enlargement with loss of fascicular pattern. Hyperintensity in muscles due to denervation.	Hypoechoic fusiform lesion in continuity with the nerve with loss of fascicular architecture with altered echogenicity of denervated muscles.
Neurotmesis	V	Rupture of the nerve	End neuroma formation at proximal end with denervation changes in muscle	Hypoechoic neuroma at proximal end with local soft tissue oedema and denervation changes in muscle.
	Mackinnon and Dellon type VI	Mixed injury	Variable findings with nerve heterogeneity and muscle denervation changes	Hypoechoic enlarged with mixed findings of scarring, discontinuity or neuroma formation.

The emerging role of imaging in peripheral neuropathy is to identify the abnormal nerve, the cause and exact site of the pathology like presence of entrapment due to any cause such as masses, anomalous muscles, fibrous bands and osseous deformities, or to show secondary findings that confirm or support the diagnosis [8]. With advances in technology and imaging sciences, high-resolution ultrasound and magnetic resonance imaging (MRI) have come in vogue for evaluation of peripheral nerve diseases. These imaging modalities provide invaluable information which is supplementary to clinical and electrodiagnostic findings [3]. These technologic developments have commenced a multimodality approach to peripheral nerve disorders. Imaging provides irreplaceable anatomical information while functional information is derived from electrodiagnostic studies [9].

Electrodiagnostic studies lack precise anatomic delineation and cannot reliably determine the underlying cause of nerve injury, making these major limitations of this modality [11]. Amongst the imaging modalities, high-resolution ultrasound (HRUS) and magnetic resonance (MR) neurography provide excellent soft tissue details and these characteristics have been exploited in visualising the pathological peripheral nerves.

The benefits of high-resolution ultrasonogram (HRUS) over MR imaging include higher soft-tissue resolution, cost effectiveness, portability, real-time and dynamic imaging and the ability to scan an entire extremity quickly and efficiently. HRUS can be performed on patients who are MR incompatible. Ultrasound is however, operator-dependent, requiring specific training and experience [11].

MRI visualises nerves, characterises soft tissue structures when evaluating atypical sites of compression, identifies features of malignancy in peripheral nerve tumours and provides information on the presence of muscle denervation and atrophy. MRI can describe nerve lesions in areas that are difficult to localise using electrodiagnostic studies or visualise using ultrasound [12]. Peripheral nerves with pure sensory function pose an imaging challenge because of their small calibre (sometimes < 1 mm [13]) and lack of associated muscle denervation, which can be seen with motor nerve abnormalities [14].

With this study, we aim to assess the accuracy of MRI and HRUS in diagnosing peripheral nerve pathologies, taking electrodiagnostic nerve conduction velocity as the gold standard. In this study, we have evaluated the peripheral nerves supplying the upper and the lower limb, namely, median nerve, ulnar nerve, radial nerve, sciatic nerve, tibial nerve, common peroneal nerve and their major branches.

## Materials and methods

### Patient population

A prospective, hospital-based study was performed over a period of 11 months, from February 2018 to December 2018 after taking ethical approval on a heterogenous group of 131 patients and 180 peripheral nerves. Consenting patients with clinical suspicion of peripheral mononeuropathy referred for ultrasound and MRI examination at a tertiary hospital without any contraindications to MRI. Any patient with symptoms, neurological assessment or radiological imaging (plain radiograph or MRI) suggestive of plexopathy or cord injury was excluded from the study. Electrodiagnostic findings (NCV) of each patient were recorded which was done by a technician with 8 years’ experience.

Data analysis was carried out using the commercially available SPSS program, Version 20.0.

### Image analysis technique

**Fig. 1:**
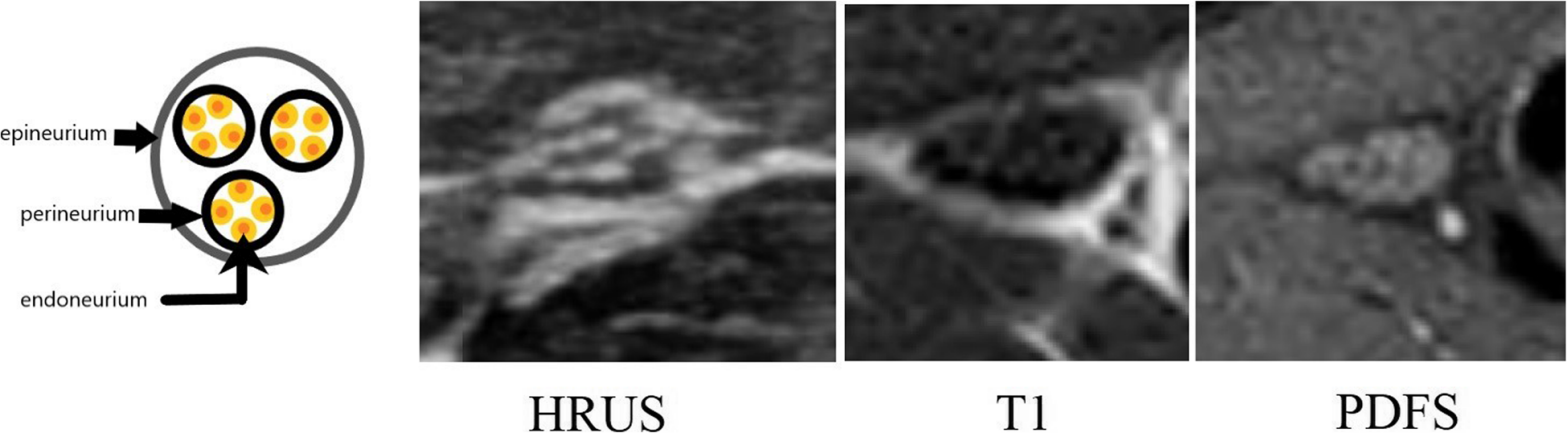
Diagrammatic representation of the neuronal architecture with the neurons (orange dots) enclosed in an endoneurial sheath. The axons, Schwann cells and endoneurium are bundled together into fascicles, each of which is encompassed by a dense perineurial sheath. The epineurium is the outermost connective tissue sheath. This results in a characteristic honeycomb pattern which is identified on HRUS and MRI in transverse and axial sections respectively

Peripheral nerve has a characteristic honeycomb appearance on ultrasound, reflecting the fascicular composition of nerve on histopathology. Nerves can be identified in the transverse section at pre-determined landmarks and then followed proximally and distally to identify the site of pathology. Normal nerves tend to have a higher echogenicity when compared to the adjacent muscles and lesser when compared to the tendons. The pathological segment of the nerve will show decreased echogenicity, loss of the normal honeycomb structure, discontinuity of nerve, focal enlargement or neuroma formation. With imaging abnormalities in the surrounding soft tissue like compressive lesions, oedema and joint effusions can also be identified. We used a 14 Hz linear transducer for ultrasound examination and recorded the total time taken in each examination. Longitudinal scans were done to ascertain continuity and contour of the nerve and compare the thickness, while the area and echogenicity was determined on the transverse scans (Fig. 1a). All ultrasound examinations were done by the same radiologist using the same equipment having more than 6 years’ experience in the field. NCV findings of all patients were not disclosed to the operator before the examination [8, 11, 12, 14].

On the same day, a focused MRI using a 3T scanner, depending upon the suspected nerve involved was carried out with the protocol elaborated in Table 2. Due to a resource-limited setting, body coils were used in all settings. Gadolinium-based contrast (Gadodiamide at 0.2 mmol/kg) was used for assessment of enhancement if a neuroma was identified or in the presence of soft tissue masses in the course of the nerve of interest. A honeycomb pattern, similar to that on ultrasound, is visible on axial sections of high-resolution MRI (Fig. 1a). Anatomical map of the concerned nerve must be well versed in order to identify it in its short axis. Normal nerves have a hyperintense peripheral rim which corresponds to the fat in the nerve sheaths and hypointense ‘dots’ which correspond to the fascicles when seen in the short axis. This allows easy identification of nerves. Pathologies are identified by increased intraneural signal on T2 images. Fat-suppressed sequences are imperative to allow identification of this high signal which can easily be masked by the epineural or perineural fat [15, 16]. Focal enlargement, hyperintensity and altered fascicular patterns are signs of nerve pathology. The denervated muscles first begin to appear hyperintense on T2-weighted images only 48 h after the nerve injury. This is seen in axonotmetic and neurotmetic type of injuries. Disruption injuries cannot be reliably differentiated from contusion of the nerve on MRI [17]. When there is failure of regeneration of the fascicles, muscle atrophy and persistent hyperintensity is seen [16] while fatty infiltration is seen in chronic denervation. Susceptibility artefacts due to orthopaedic implants limit the use of MRI to some extent. The MR images in our study were read by another radiologist, with 9 years’ experience, blinded to the findings of ultrasound and NCV.

**Table 2:** 3T MRI protocol Used In The Study With A 10% gap and FOV not more than 150–160 cm

MR sequence	TR (ms)	TE (ms)	Slice thickness (mm)	Matrix
Axial T1	748	20	3	256 × 384
Axial T2	5135	100	3	256 × 384
Axial PD FS	3088	30	3	256 × 256
Coronal/Sagittal T2	5135	100	3	256 × 384
Coronal/Sagittal PD	3088	30	3	256 × 256

Presence or absence of neuroma, defined as a focal fusiform nerve swelling, was documented for each examined nerve. Neuroma can either be a pseudoneuroma or a true neuroma, both of which can be reliably differentiated by imaging studies. Compression or entrapment of the nerve corresponds to grade III Sunderland injury results in focal nerve enlargement known as pseudoneuroma. In its true sense, neuromas refer to nerve scarring with fibrosis and resultant loss of fascicular architecture, which in Sunderland’s terms corresponds to a grade IV injury. An intact intraneural architecture in an enlarged nerve is seen on HRUS and MRI in a pseudoneuroma while discontinuity of fascicular architecture is seen in a true neuroma [20, 21].

Thorough understanding of the anatomical relations of the target nerve is of utmost importance to identify the nerve and possible aetiology for the underlying pathology. Additional file 1 provides detailed anatomical information along with relevant figures to identify the nerves included in the study.

## Observation and results

### Demographics

**Fig. 2:**
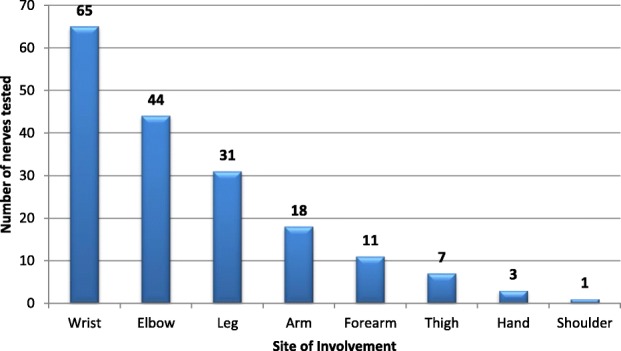
Diagrammatic representation of the site of nerve pathology

In our study, majority (28.24%) of the subjects belonged to the age group of 31–40 (Table 3) with a male to female ratio of 1.42:1. The median nerve was the most common nerve tested, comprising of 35.56% of total nerves tested (Table 4) while wrist was the most common site examined for neuropathy (36.11%) followed by the elbow (24.44%) and leg (17.22%) (Fig. 2). Overall, the most common aetiology of neuropathy was carpal tunnel syndrome (45 cases) while post-traumatic neuropathy was diagnosed in 37 cases.

**Table 3:** Distribution of study subjects according to the age group

Age groups (in years)	Number	Percentage
< 10	4	3.05
11–20	6	4.58
21–30	35	26.72
31–40	37	28.24
41–50	24	18.32
51–60	14	10.69
61–70	8	6.11
> 70	3	2.29
Total	131	100.00

**Table 4:** Distribution of the peripheral nerves examined in the study

Name of nerve tested	Number	Percentage
Median nerve	64	35.56
Ulnar nerve	54	30.00
Common peroneal nerve	23	12.78
Radial nerve	15	8.33
Sciatic nerve	7	3.89
Tibial nerve	7	3.89
Posterior interosseous nerve	5	2.78
Superficial branch of radial nerve	3	1.67
Superficial peroneal nerve	1	0.56
Supraspinatus nerve	1	0.56
Total	180	100.00

### Diagnostic confidence

Out of the 180 nerves tested, 150 tested positive on the gold standard, electrodiagnostic study (NCV) while 30 were negative on NCV. HRUS detected 137 of the positive cases while MRI proton density (PD) fat-saturated (FS) sequence detected 147 of the total positive cases. All 137 cases detected by HRUS were also detected by MRI except one in which the superficial branch of the common peroneal nerve was entrapped in the superficial fascia of the leg resulting in compressive neuropathy. Probably due to the millimetric size of the nerve, the alteration in signal intensity was not well perceived on MR while HRUS depicted focal change in calibre and echogenicity.

The diagnostic confidence of PD FS MR sequence was seen to be highest with a sensitivity of 95.34%. Only in seven out of 150 positive nerves, PD FS MR sequence failed to detect the abnormal nerve. T2 MR sequence showed a sensitivity of 73.33%. The sensitivity of HRUS in our study was 87.33%. HRUS could not identify the abnormal nerve in 19 out of 150 cases.

T1 MR sequence had the highest specificity of 96.67% followed by the T2 and PD FS sequences which had equal specificity of 86.67% each. HRUS was the least specific with a specificity of 80%. The positive predictive value was highest for T1 MR sequence (98.48%) while HRUS had the least positive predictive value (95.62%). A 78.79% negative predictive value was calculated for MRI PD FS sequence while T1 MR sequence had the lowest negative predictive value of only 25.44%.

**Fig. 3:**
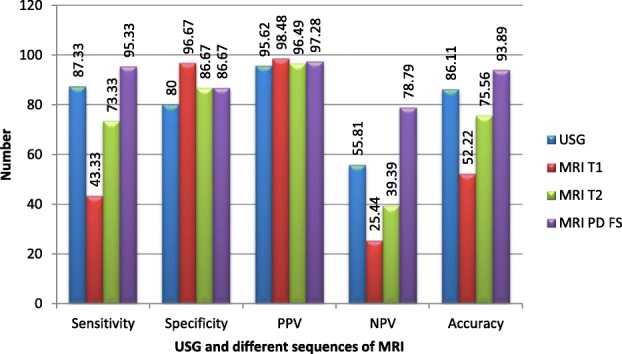
Diagnostic value of HRUS and different sequences of MRI on the basis of the study findings

Overall, the accuracy was highest for PD FS MR sequence (93.89%) followed closely by HRUS (80%). These findings are summarised in Fig. 3. The combined accuracy of both modalities was calculated to be 93.33% implying that MR examination with PDFS sequence alone will identify most pathological nerves. HRUS is more widely available, cost effective, quicker, feasible for evaluation of patients with MR unsafe implants and has a comparable diagnostic accuracy. These properties of HRUS can be employed in screening patients with suspected nerve pathologies.

### Continuity of nerve

In our study, six out of 37 cases of post-traumatic neuropathy had nerve discontinuity, all of which were diagnosed by ultrasound and MRI. In the remaining causes of non-traumatic neuropathy (infective, inflammatory, mass lesion, unclassified [24]), continuity was seen in 100% nerves. On comparing MRI and HRUS in assessment the continuity of the nerve, both were equally sensitive (100%) when compared to the surgical findings (obtained in 28/37 patients).

### Neuroma formation

Neuroma formation was seen in 25 cases on MRI out of which HRUS did not detect three cases correctly. HRUS could detect 88% nerves with neuroma when compared with MRI (100%). Eighteen out of the 25 cases diagnosed to have a neuroma on imaging testified the same on surgical exploration. The remaining patients refused surgery.

### Muscle changes

Fifteen affected nerves had concomitant altered muscle intensities on MRI while HRUS detected increase in muscle echogenicity in only four cases.

### Time taken for HRUS and MRI

MRI, on an average, took five times longer for a complete nerve study when compared to HRUS. Overall, the mean time taken for a complete MRI study was 37.32 min with a standard deviation of 5.36. HRUS required considerably less amount of time with a mean of 7.91 min and standard deviation of 2.05 min.

### Aetiology of nerve pathology in our study

**Fig. 4:**
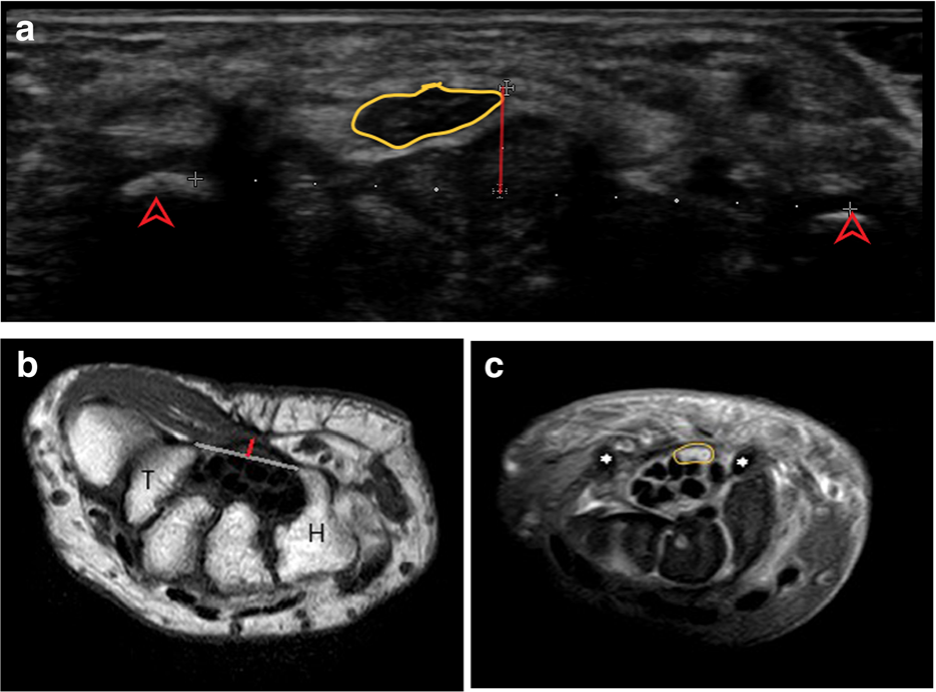
**a** Median nerve is hypoechoic (yellow outline) at the level of the carpal tunnel inlet (arrowhead) with bowing of the flexor retinaculum (red line) at the inlet measuring 3.4 mm in this patient with CTS. **b** Axial T1-weighted MR image at the carpal tunnel outlet (T- trapezium, H- hamate) shows bowing of the flexor retinaculum (red line), 2.8 mm in this patient with CTS. **c** Hyperintense median nerve (yellow outline) is seen on PD FS axial MR image at the carpal tunnel inlet (asterix) in the same patient with CTS

Idiopathic carpal tunnel syndrome (CTS), the most common aetiology diagnosed in our study, has been diagnosed, like all other neuropathies, conventionally by NCV testing. Table 5 summarises the findings in CTS with reference cut-off values for all the parameters assessed in this study. One added advantage of HRUS over MRI in this clinical entity is dynamic scanning. HRUS allows examination of the median nerve both at rest and during flexion of the wrist. In normal subjects, there is smooth translation of median nerve towards ulnar side on complete flexion with return to neutral position on extension. This movement is restricted in patients with CTS and is one of the parameters, albeit subjective, assessed by us in our study [25] (Fig. 4a–c).

**Table 5:** Parameter for evaluation of carpal tunnel syndrome [18, 19]

Parameter	Method	USG reference value			MRI reference value		
		DRUJ	PISIFORM	HAMATE	DRUJ	PISIFORM	HAMATE
CSA (mm^2^)	Continuous trace method	7.55 (1.58)	10.3 (2.3)	9.00 (1.98)	9.0 (2.5)	9.1 (2.3)	8.8 (1.8)
Flattening ratio	Transverse axis divided by longitudinal axis	2.2 (0.29)	2.27 (0.3)	2.96 (0.4)	2.38 (0.61)	2.62 (0.55)	2.76 (0.75)
Palmar displacement (mm) (measured on T1 image)	Perpendicular distance to the TCL from the line between the hook of the hamate and the trapezium		2.9	2.5			2.58 (0.82)
Swelling ratio	Middle CSA to proximal CSA		1.3				

*CSA* curved surface area (measurements done on PD FS image), *DRUJ* distal radio ulnar joint

All measurements were done at three levels, at the level of the distal radio ulnar joint, level of pisiform bone and lastly at the level of the hook of hamate

**Table 6:** Comparative analysis with existing literature

Study	Nerves studied	Sensitivity		Specificity		Accuracy		MRI strength
		USG	MRI	USG	MRI	USG	MRI	
Andreisek	51		93					
Zaidman	46	93	67					1.5T
Garg	64	81.25	95.31 (T2)					3T
Our study	180	87.33	95.33 (PDFS)	80	86.67 (PDFS)	86	93.89 (PDFS)	3T

**Fig. 5:**
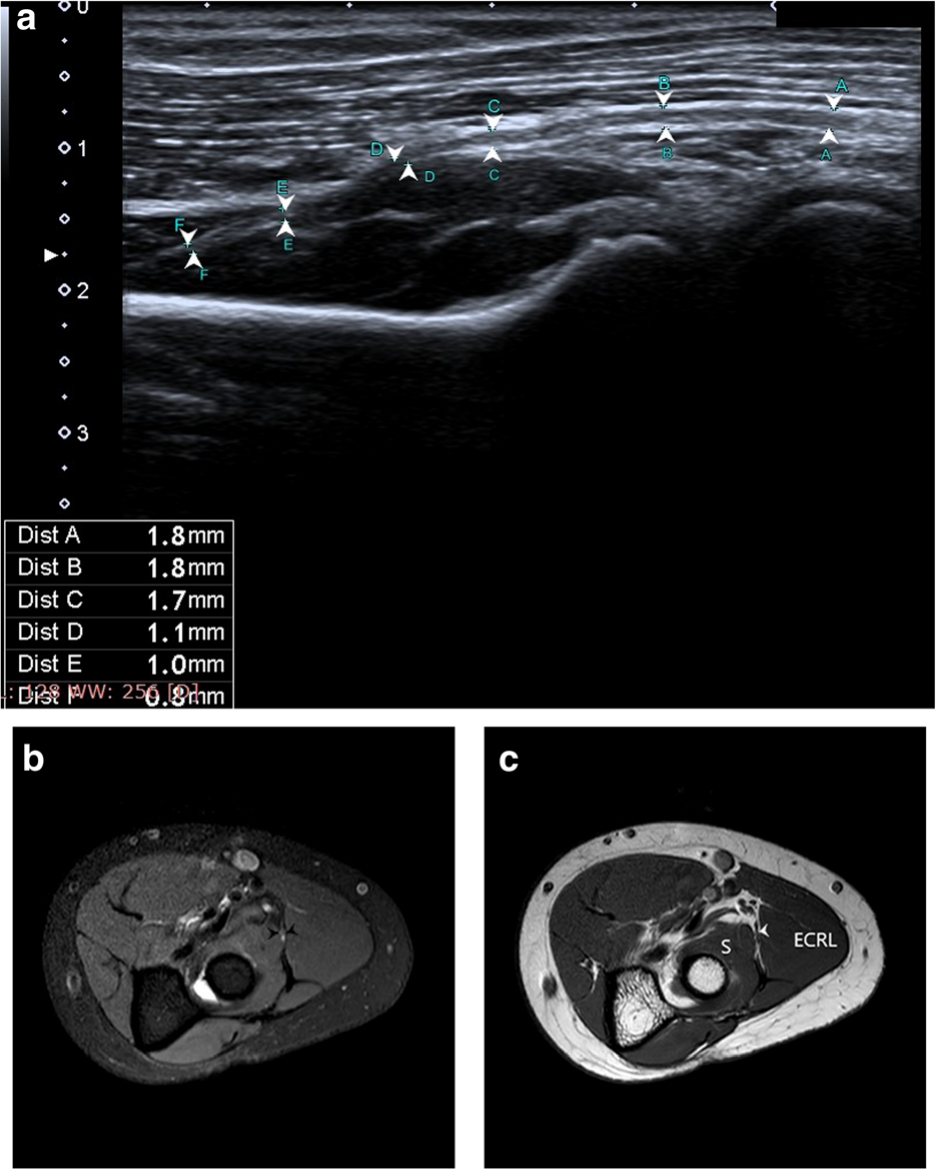
**a** Longitudinal scan over the radial head shows the PIN (between arrowheads) coursing over the radial head and passing through the radial tunnel. The nerve is hypoechoic and thickened just before passing through the arcade of Frohse in a patient with radial tunnel syndrome. **b** PD FS axial image shows hyperintense PIN with bulbous swelling just before it pierces the arcade of Frohse, lying between the distal edge of ECRL and superficial supinator. **c** T1-weighted axial image shows the course of the PIN soon after its division from the radial nerve passing between the ECRL and supinator. The diagnosis of radial tunnel syndrome was confirmed in this patient by giving ultrasound-guided PIN block [22]

**Fig. 6:**
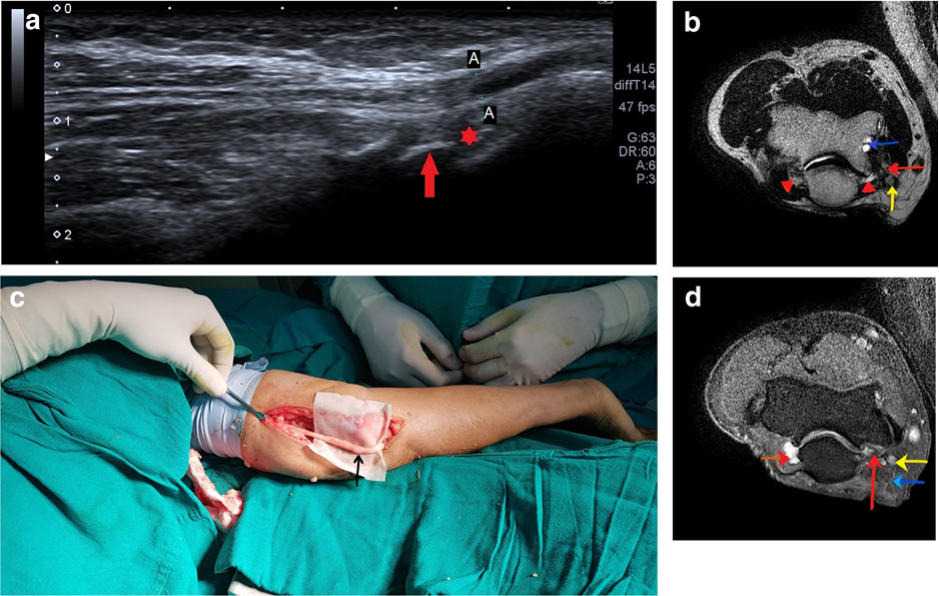
**a** Hypoechoic ulnar nerve (A) is seen at the level of the cubital tunnel with underlying synovial thickening (*) and cortical irregularity (red arrow). **b** T2-weighted MR image shows thickened hyperintense ulnar nerve (yellow arrow) with surrounding synovial thickening (red arrowhead) and joint effusion. Note is made of subchondral cysts (blue arrow) and cortical irregularity (red arrow). **c** A hyperintense ulnar nerve (yellow arrow), joint effusion (red arrow) and panniculitis (blue arrow) can be seen behind the median epicondyle in the cubital tunnel on this PD FS image. Chronic inflammation has led to cubital tunnel syndrome in this patient. **d** Intra-operative picture of the patient with cubital tunnel syndrome due to chronic inflammation shows thickened ulnar nerve

Apart from this, radial tunnel syndrome (Fig. 5a–c) and cubital tunnel syndrome (Fig. 6a–d) were the other entrapment syndromes encountered in our study.

**Fig. 7:**
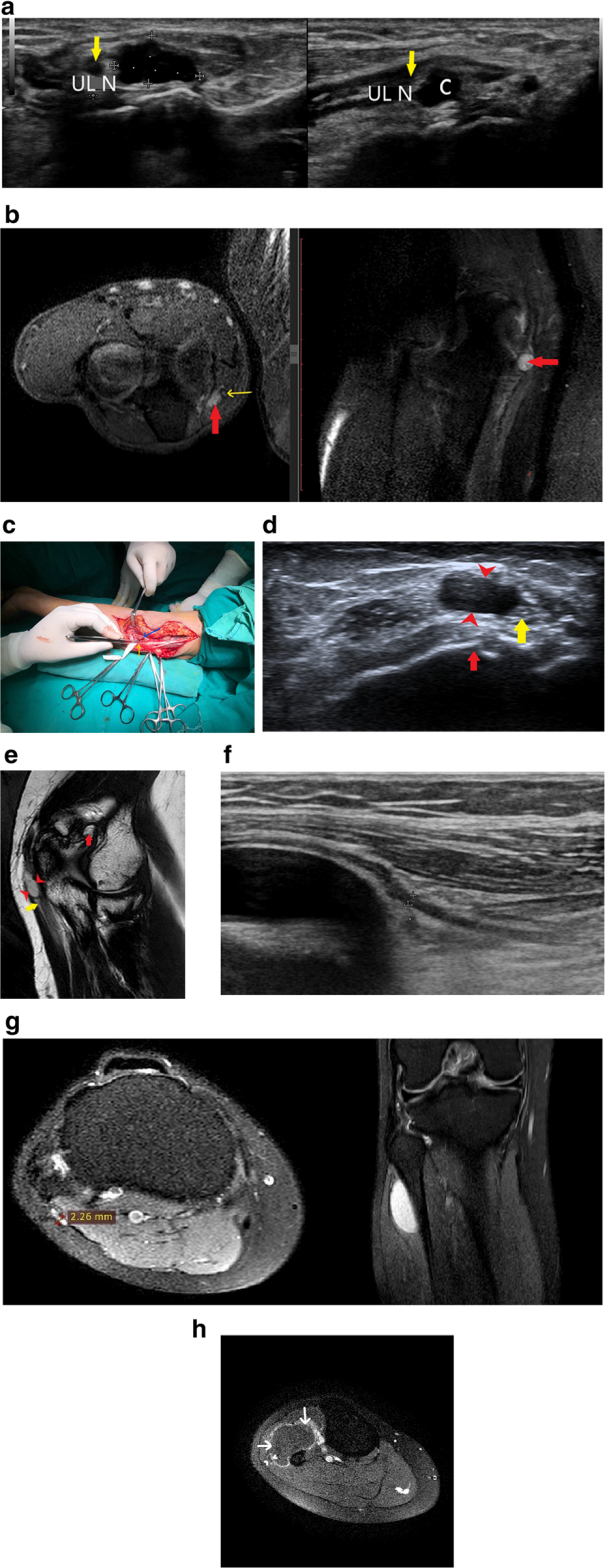
**a** There is a cystic lesion (between callipers) causing direct compression over the ulnar nerve (yellow arrow) which appears hypoechoic. **b** PD FS axial and sagittal image demonstrate a hyperintense ulnar nerve (yellow arrow) which is getting compressed by a hyperintense cystic lesion (red arrow). **c** Per operative picture showing the ulnar nerve (yellow arrow) and the cyst (blue arrow). Histopathology revealed it to be a ganglion cyst. **d** Anechoic cystic lesion (red arrow heads) adjacent to the ulnar nerve (yellow arrow) at the elbow is seen with underlying cortical erosions (red arrow). The cystic lesion appears to arise from the nerve, suggestive of an intraneural ganglion cyst. **e** The hyperintense cystic lesion (red arrow heads), as seen in 5D, is seen arising from the adjacent hyperintense ulnar nerve (yellow arrow) lying along the course of the nerve on T2-weighted image [23]. Also noted are osteophytes (red arrow). **f** High-resolution ultrasound image showing common peroneal nerve (arrowheads) lying stretched over the underlying cystic lesion. **g** Hyperintense CPN is noted in this axial PDFS image (between crossheads) just before it wraps around the neck of fibula. The nerve is hyperintense as it is passing over the underlying cysts as seen on the right. PDFS image on the right shows hyperintense cysts which on excision biopsy proved to be ganglion cysts. **h** T1-weighted fat saturated post-contrast image shows enhancing cyst walls with no internal enhancement (arrow) suggestive of cystic nature of the lesion. Also seen is enhancement of the sheath of the common peroneal nerve (arrowhead) lying in close proximity to the cyst

**Fig. 8:**
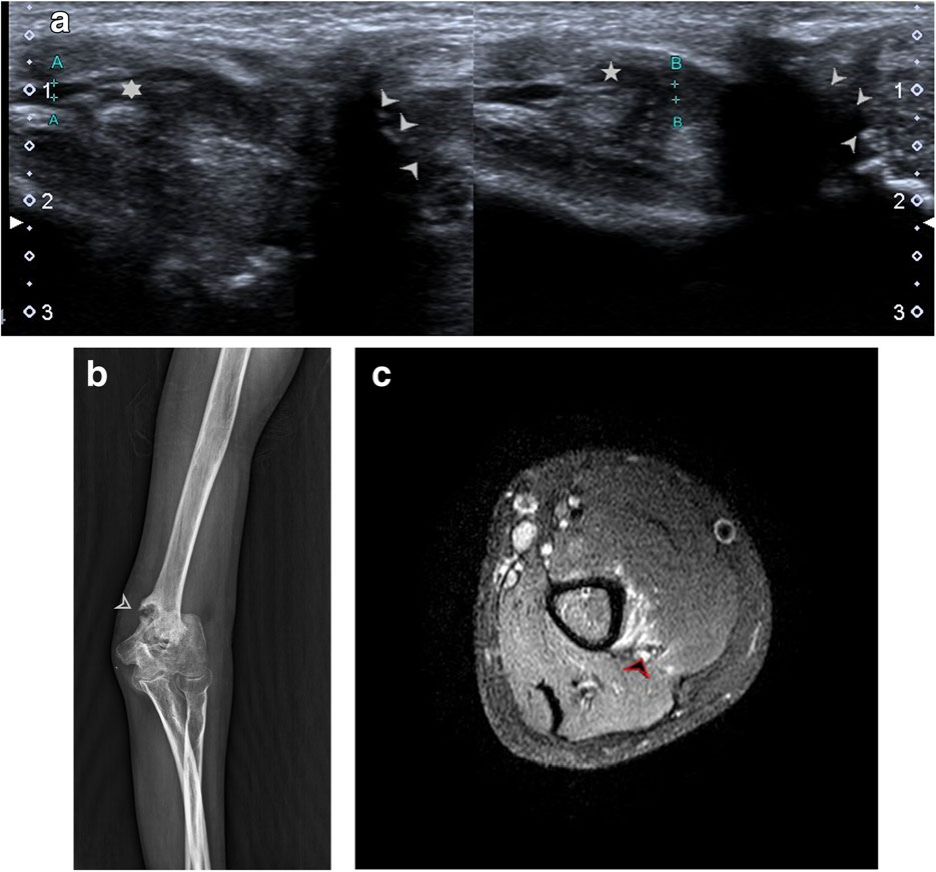
**a** Hypoechoic radial nerve (point A and B) is seen entrapped within mixed echogenic chronic inflammatory soft tissue (5 point and 6 point star). Malunited fracture of humerus can be seen adjacent to it (arrowheads). **b** Plain radiograph showing malunited, displaced fragment of supracondylar fracture of humerus. **c** Hyperintense radial nerve (black arrowhead) with surrounding hyperintense muscle oedema can be seen on this axial PDFS image

**Fig. 9:**
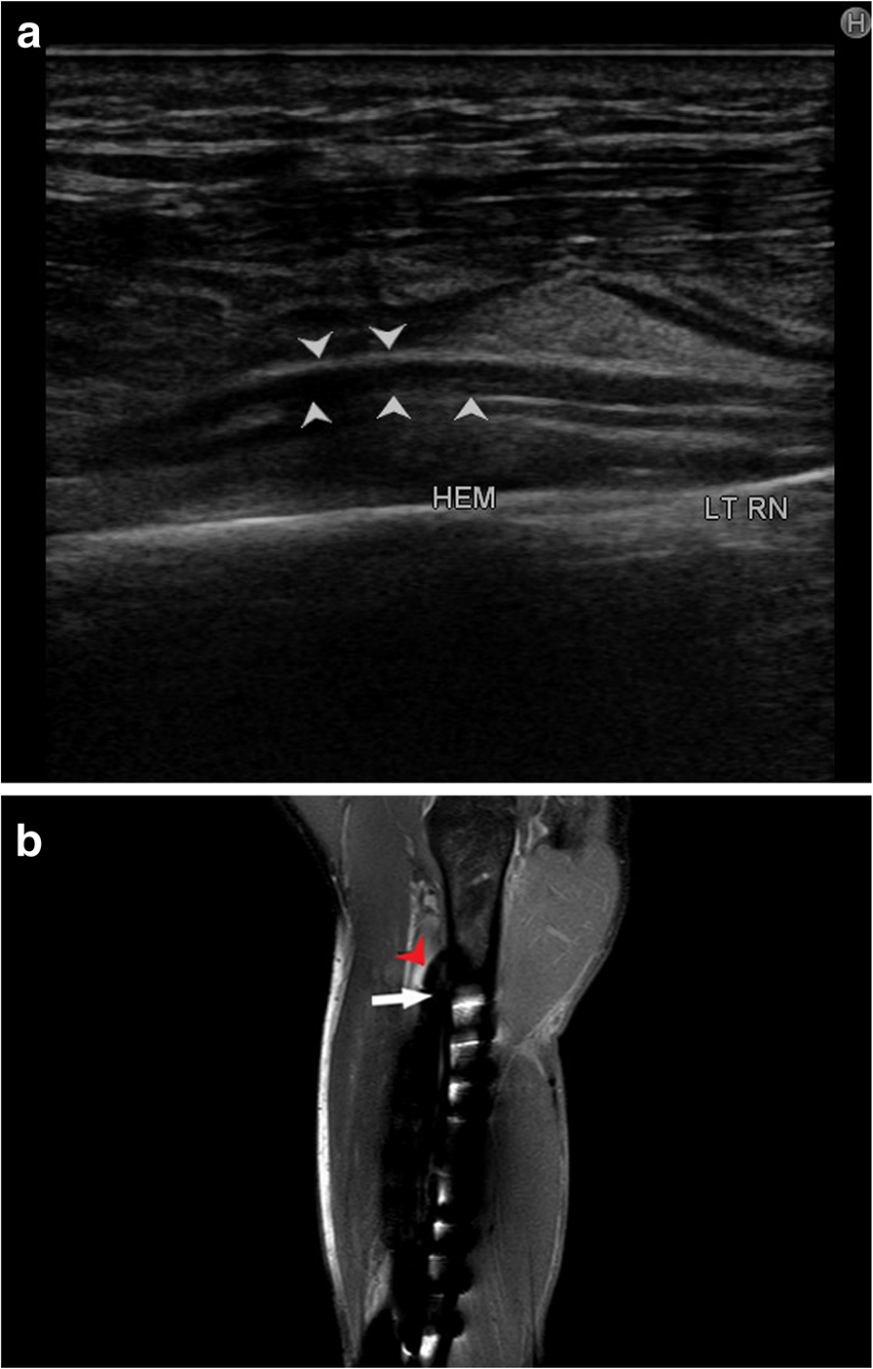
**a** Long axis view of the left radial nerve (arrowhead) showing displacement by local hematoma (HEM). **b** Coronal PDFS sequence showing hyperintense radial nerve (black arrowhead) with underlying hematoma in acute stage, as seen in (**a**) is demonstrated. Hyperintense screws of the medullary nail implanted for fixation of the fracture of shaft of humerus can also be seen

**Fig. 10:**
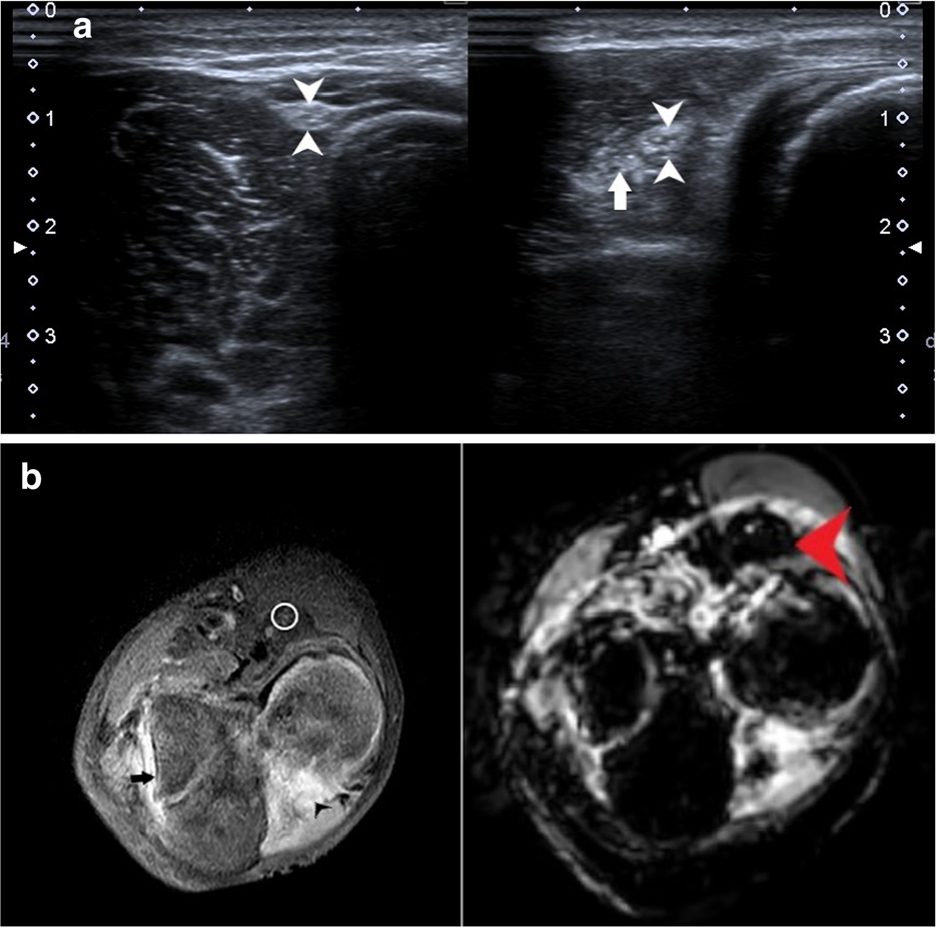
**a** Normal radial nerve (between white arrowheads) at the level of the spiral groove on the left with a mildly hypoechoic radial nerve surrounded by echogenic soft tissue (white arrow) in the distal arm as seen in the panel on the right. Based on ultrasound findings, radial nerve entrapment in scar tissue or hematoma was suspected. **b** The radial nerve (encircled), just before its division into the superficial and deep components is hyperintense on PDFS image with surrounding hypointense soft tissue. There are also changes of haemophilic arthropathy as evident by the hyperintense joint effusion (arrowheads), cortical irregularities and cartilage thinning (black arrow). On corresponding SWI image, there is blooming in the area around the radial nerve, which corresponds to the echogenic soft tissue on ultrasound (red arrowhead). This was a case for radial neuropathy due to entrapment in local hematoma in a case of haemophilia

Compressive neuropathies include nerve compression by perineural or intraneural ganglion cysts (Fig. 7a–h). Post-traumatic compressive neuropathies can also result due to entrapment in local scar tissue, inflammatory tissue (Fig. 8a–c), compression by a hematoma (Fig. 9a, b) or entrapment in clot, as in one case of a patient with haemophilia (Fig. 10a, b).

**Fig. 11:**
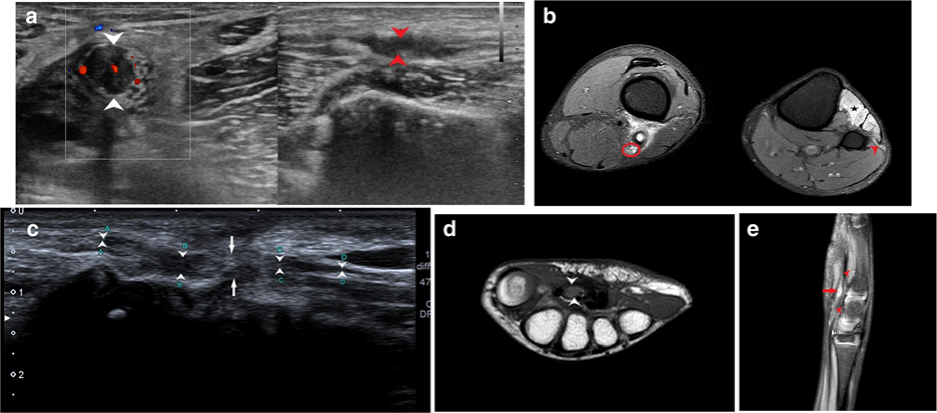
**a** Transverse HRUS shows partial loss of fascicular architecture with nerve thickening in the CPN (white arrowheads in panel on the left) soon after its origin from the sciatic nerve in a patient with post-traumatic foot drop with concomitant osteomyelitis. Also note the increased vascularity in the nerve suggestive of neuritis. The CPN was discontinuous with formation of this ‘end neuroma’. The panel on the right shows the distal segment of the discontinuous CPN which is hypoechoic and thickened (red arrowheads). **b** PDFS MR axial image shows pseudoneuroma with partially preserved fascicular architecture (encircled in red) with surrounding oedema, with distal segment showing hyperintensity (red arrowhead). There is hyperintensity in the peronei muscles (asterisk) suggestive of failure of regeneration. **c** Thickened hypoechoic median nerve (between arrowheads) with loss of continuity (arrow) in a case with penetrating injury to the forearm. **d** Axial T1-weighted image shows loss of fascicular architecture with hypointense neuroma formation (arrowheads) at the distal cut end of the proximal segment. **e** Sagittal PDFS image shows hyperintense median nerve (arrowheads) with focal loss of continuity which is seen as linear hyperintensity (red arrow) in the same patient

**Fig. 12:**
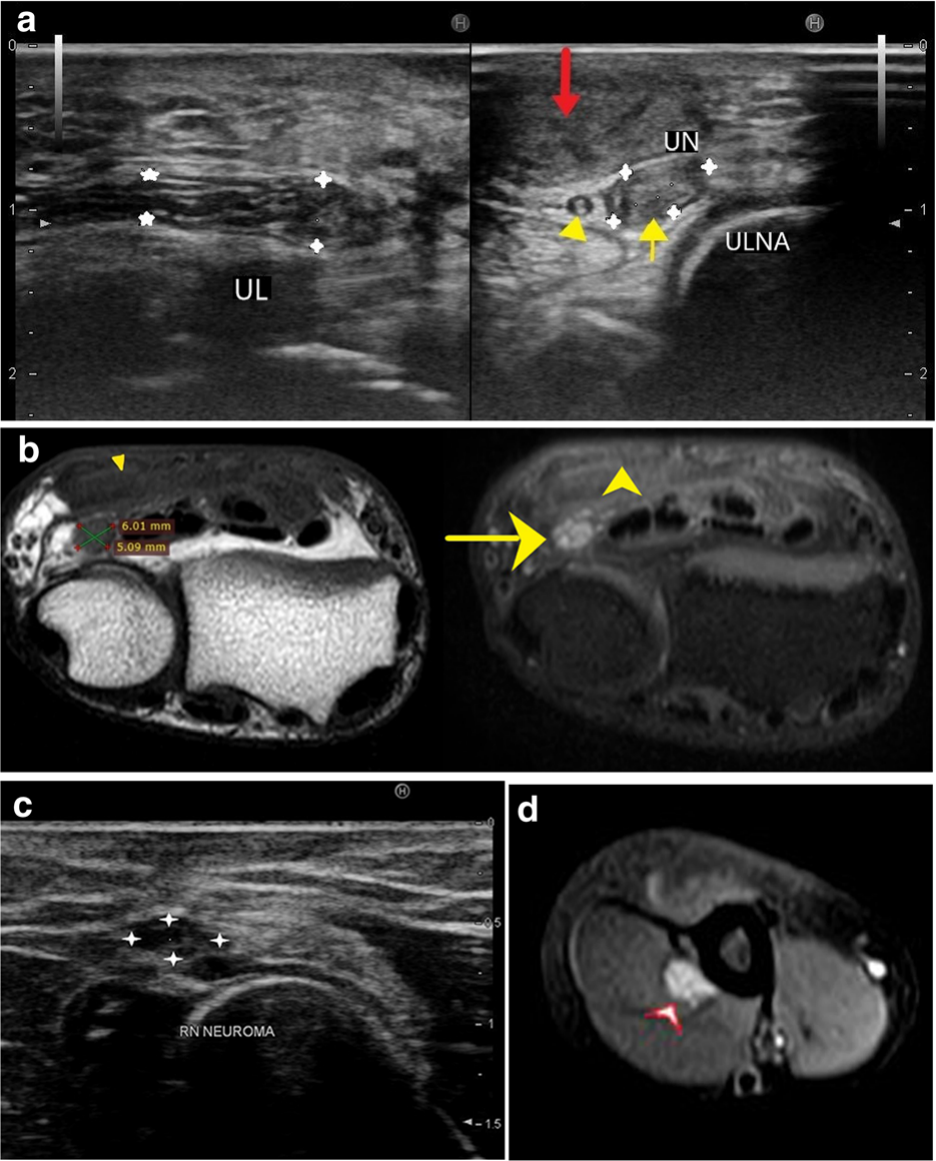
**a** In a case with glass cut injury at the wrist, a hypoechoic ulnar nerve (between arrowheads) is noted with formation of neuroma in continuity (yellow arrow) characterised by thickened, hypoechoic focal area with loss of fascicular architecture. Also noted is soft tissue swelling (red arrow). Yellow arrowhead marks the ulnar artery. **b** T1- and PDFS-weighted images show the ulnar nerve neuroma which is hypointense on T1 and hyperintense on PDFS (between callipers and yellow arrow respectively) with associated soft tissue oedema (arrowhead). **c** Focal thickening suggestive of radial nerve neuroma (between cross marks) is seen distal to the spiral groove in a case with monkey bite at the arm. The nerve is seen lying between the brachialis and brachioradialis. **d** Axial PD fat-saturated sequence of the same patient demonstrates a well-defined ovoid hyperintense lesion (black arrowhead) in continuity with the radial nerve. As the lesion was noted distal to the spiral groove, after supply to the triceps, there is no signal intensity change in the triceps muscle signifying normal innervation to the muscle

**Fig. 13:**
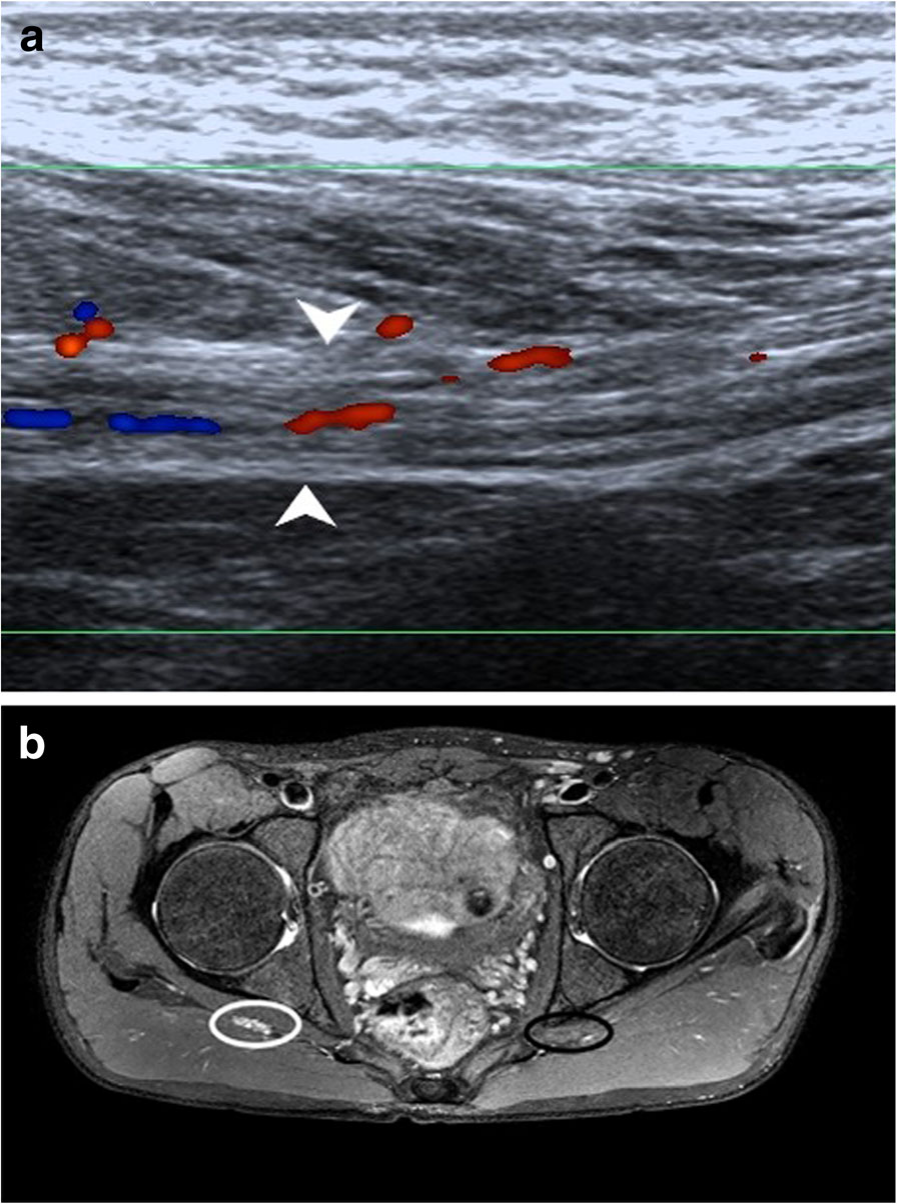
**a** Sciatic nerve (between arrowheads) is thickened with increased vascularity suggestive of neuritis in a patient with post-injection palsy. **b** PDFS axial image shows hyperintensity in the right sciatic nerve (white circle) with opposite normal sciatic nerve (black circle)

Penetrating injuries can cause direct trauma to the nerves resulting in various grades of neuropathy as described by Sunderland and Seddon (Table 1). This may lead to a loss of continuity of the nerve with end neuroma formation (Fig. 11a–e), formation of a neuroma in continuity (Fig. 12a–d) or simply hyperintensity (Fig. 13a, b) depending upon the extent of the injury.

**Fig. 14:**
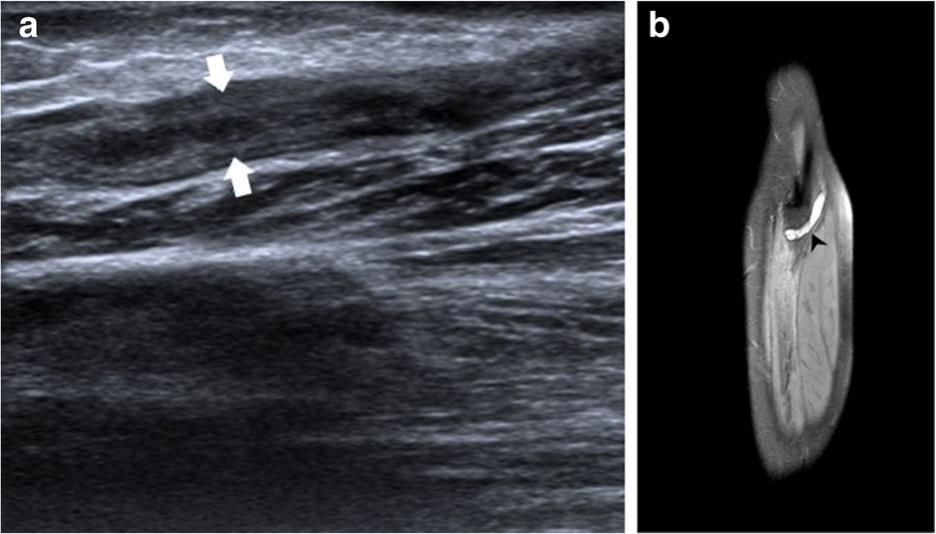
**a** Longitudinal scan of ulnar nerve at the elbow (arrowheads) shows a thickened nerve with loss of fascicular architecture. There was also increased vascularity suggestive of neuritis in this patient with Hansen’s disease. **b** PDFS sagittal image shows a tubular hyperintense CPN (arrowhead) as it winds around the fibular neck in a case with Hansen’s disease. There is long segment thickening, loss of fascicular architecture with incidentally detected high division of the sciatic nerve. There was also associated fatty infiltration with muscle hyperintensity due to chronic denervation

Leprotic involvement of peripheral nerves is not unheard of and may manifest in a variety of ways. It may present as an intradermal nerve involvement in a visible cutaneous patch or may occur as an isolated lesion in the distribution of the affected nerve [26]. The patient may or may not have neurogenic symptoms. On imaging, the nerve may appear hypoechoic with loss of fascicular architecture or have frank abscess formation, cystic change with intraneural hypervascularity (Fig. 14a, b).

**Fig. 15:**
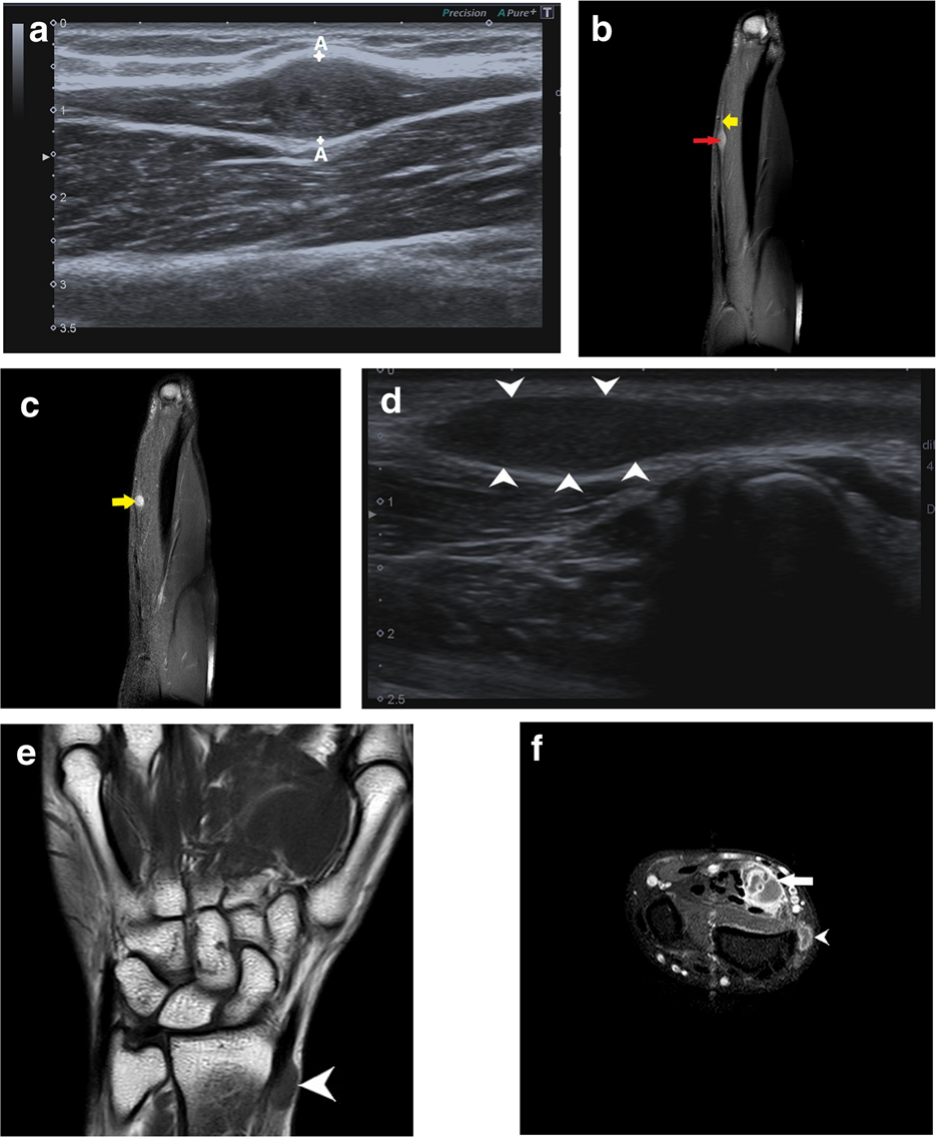
**a** Characteristic fusiform swelling in continuity of the ulnar nerve can be seen on this ultrasound image, suggestive of peripheral nerve sheath tumour. **b** PD FS image shows a fusiform swelling which is hyperintense (red arrow) with internal hypointense areas, suggestive of collagenous stroma. The ulnar nerve (yellow arrow) can be seen forming a tail, entering and exiting from the lesion, which was diagnosed to be a PNST. **c** Post-contrast T1 FS image shows vivid enhancement in the PNST. **d** Well-defined fusiform hypoechoic lesion (white arrowheads) is noted in continuity of superficial branch of radial nerve in the region of the anatomical snuffbox, just distal to the radial styloid. **e** Similar lesions are seen on T1-weighted coronal image. A well-defined, T1 hypointense, fusiform lesion (white arrowhead) in continuity with the superficial branch of radial nerve at the distal end of radius can be seen. **f** Axial post-contrast fat-saturated T1 image showing well-defined hypointense lesion surrounded by an enhancing capsule (arrowhead) in continuity with superficial branch of radial nerve. Another similar lesion (arrow) showing heterogenous contrast enhancement with hypointense areas within can be seen in continuity with the median nerve. This lesion was T2 and PD hyperintense with cystic degenerative changes on ultrasound and MRI. These MR findings fit the description of ancient schwannomas [35]

PNSTs, either benign or malignant, include two entities—neurofibroma and schwannoma. These may present as soft tissue masses or may be incidentally detected depending upon their location, superficial or deep. PNSTs usually have a normal plain radiograph or may show enlargement of the soft tissue shadow. On ultrasound, a hypoechoic fusiform mass may be seen in continuity with the parent nerve which points towards its neurogenic origin. Differentiation between the two main differentials, PNST and neuroma is possible by MRI. On MRI, a T1 iso-hypointense, T2 hyperintense fusiform mass is seen in continuity with the parent nerve with central low signal on T2WI. This central T2 hypointensity corresponds to the higher fibro-collagen content which also co-relates with the organised histological architecture and is known as the ‘target sign’. Another sign is the split fat sign wherein the hyperintense fat on T1 images around the fusiform mass suggests its intermuscular location. Detection of fascicular pattern within the mass also supports its neurogenic origin [27]. Literature review shows discrepancy in the utility of post-contrast scans to differentiate between PNST and neuromas. While some [27] suggest that enhancement is seen in PNST and not in neuromas, others like Ahlawat et al. suggest that due to a broken blood-brain barrier, even 88% of traumatic neuromas are enhance. According to them, the absence of target sign and history of trauma are the most reliable signs of a traumatic neuroma [28] (Fig. 15a–f).

## Discussion

In our study of 180 peripheral nerves with suspected peripheral neuropathy, ultrasound had a fairly high sensitivity of 87.33% with an accuracy of 86.11%. HRUS, on an average, was five times quicker in evaluating a nerve when compared to MRI. MRI, with better soft tissue contrast also has the advantage of multiple sequences which help in better characterisation of the lesion. The T1 MR sequence had a specificity as high as 96.67% while PDFS sequence had a sensitivity of 95.33% and an accuracy of 93.89% (Fig. 3).

PD FS sequence was the best amongst the all three sequences in our study in identification of the pathological segment of the nerve. HRUS was less sensitive when compared to MRI in diagnosing neuromas but both HRUS and MRI equally identified all cases with nerve discontinuity and thickening.

All except one case of post-traumatic neuropathy were identified by HRUS, making HRUS highly useful in settings of trauma, allowing bedside, quick and reliable assessment of nerve damage. Patients with neuropraxia (Sunderland grade I) were better detected on MRI when compared to HRUS with HRUS being negative in 12% of cases of CTS and 14% of post-operative tourniquet palsy cases.

There have been studies in the past comparing the two modalities in peripheral nerve imaging (Table 6). Zaidman et al. [11] conducted a comparative study with 1.5T MRI in 53 cases and found the sensitivity of ultrasound to be higher than MRI. Our results differ from their study which might be due to inclusion of multifocal lesions in their study and a lower MR strength. The sequences which they used in the MRI scans have not been mentioned in their study. Theirs was a retrospective review of already imaged cases taking NCV or surgical findings as gold standard.

Andreisek et al. in 2000 [29] and Garg K et al. [8] did a similar study and their results match with ours. The sensitivity of MRI in the study by Andreisek was 93% while the T2 sequence of MRI in the prospective study with a similar study design by Garg K et al. had a sensitivity of 95%. In our study, PD FS MRI sequence had the highest sensitivity 95.33%.

Our study has the largest number of nerves studied when compared to the other available literature. Also, we did not find any other large-scale study comparing high-resolution ultrasound and MRI in terms of their specificity, negative predictive value and accuracy. We have calculated these parameters taking electrodiagnostic study as the gold standard. Comparison of our study with the aforementioned literature is elaborated in Table 3.

Diffusion tensor imaging and tractography are the new entrants in imaging of peripheral nerve pathologies. Diffusion tensor imaging and tractography were used by Jengojen et al. [30] to assess acute post-compressive neuropathic changes in radial and median nerves. Their study demonstrated higher vulnerability of radial nerve for compression in the spiral groove. This also reflects upon the pathogenesis of tourniquet palsy. Razek et al. [31] demonstrated a positive correlation between findings of diffusion tensor imaging, electrodiagnostic tests and clinical assessment in a prospective study in 39 patients with mild to moderate carpal tunnel syndrome. They demonstrated lower fractional anisotropy values in neuropathic segments [32]. Another study by Razek et al. [33] demonstrates use of DTI in assessment of peripheral neurogenic tumours. Kermarreck et al. [34] worked on newer prospects in peripheral nerve imaging and concluded that tractography is still emerging in the musculoskeletal field, particularly for the analysis of peripheral nerves, but this technique seems promising.

The major limitation of our study is suboptimal image quality due to non-availability of dedicated coils as ours is a resource-limited setting. All MRI examinations of the upper limb and the proximal lower limb had to be performed using a body coil and ultrasound probes with a frequency higher than 14 Hz were not available at our hospital. Newer sequences like DTI and DWI have not been incorporated in our study.

High-resolution ultrasound is a sensitive and accurate technique to diagnose peripheral nerve pathologies and HRUS can be efficiently used as a screening modality. Focussed MRI can then be performed to confirm the HRUS diagnosis and supplement its findings as MRI provides additional information about denervation changes in muscle and signal changes in the surrounding soft tissues. Further advancements like DTI and DWI can be incorporated to further improve the diagnostic accuracy. HRUS is less time consuming and more cost effective when compared to MRI.

Different sequences of MRI combined together may serve as an imaging gold standard for diagnosing peripheral nerve pathologies with HRUS being an effective screening tool. PDFS being superior to T2 can be used solely with T1 and post-contrast images if needed. This will help in reducing total study time in settings with time constrains and limited resources. These two imaging modalities are not mutually exclusive. Rather, they complement each other and can be used in conjunction as an imaging yardstick for diagnosing peripheral neuropathies.

## Additional file

Additional file 1: Anatomical details. (DOCX 5149 kb)

## Data Availability

The datasets used and/or analysed during the current study are available from the corresponding author on reasonable request.

## Abbreviations

CPN: Common peroneal nerve
CTS: Carpal tunnel syndrome
FS: Fat saturated
HRUS: High-resolution ultrasound
MRI: Magnetic resonance imaging
PD: Proton density
PIN: Posterior interosseous nerve
