# Supplementary material for: Can imaging be the new yardstick for diagnosing peripheral neuropathy?—a comparison between high resolution ultrasound and MR neurography with an approach to diagnosis
Source: Insights Imaging. 2019 Nov 1;10:104. doi: 10.1186/s13244-019-0787-6 (PMC6825074; doi:10.1186/s13244-019-0787-6)
Supplement: Supplementary file 1 — Anatomical details. (DOCX 5149 kb) [file 13244_2019_787_MOESM1_ESM.docx]

ANATOMICAL DETAILS

The basic unit of the peripheral nerve is the axon, which may be myelinated or unmyelinated, and carry efferent (motor) or afferent (sensory) electrical impulses [1]. Myelinated axons are enclosed by many layers of compacted Schwann cell membranes, which form the myelin sheath, whereas “unmyelinated” axons merely invaginate into grooves in the Schwann cell cytoplasm [2]. The largest peripheral nerves have three connective tissue sheaths that support
and protect the complex. The innermost sheath is the endoneurium. The axons, Schwann cells, and endoneurium are bundled together into fascicles, each of which is encompassed by a dense perineurial sheath. The epineurium is the outermost connective tissue sheath. This specific arrangement of nerves gives a typical honeycomb appearance which helps in identification of nerves on ultrasound and MRI in transverse scans and axial sections respectively [2,3]. [Figure 1a]

ULNAR NERVE [Figure 1b-1h]

The nerve arises from the medial cord of the brachial plexus carrying fibres from the C8-T1 nerve roots. It lies posteromedial to the brachial artery in the anterior compartment of the upper arm and descends down to enter the posterior compartment by piercing the medial intermuscular septum. This is a potential site of compression of the nerve under the ‘Arcade of Struther’ [4] which is described as a fibrous canal on the medial aspect of the middle- and lower-third of the arm, consisting of the medial head of the triceps brachii muscle and its aponeurotic expansion, which extends into the intermuscular septum and internal brachial ligament and covers part of the ulnar nerve. Thereafter, the ulnar nerve runs behind the medial epicondyle with the superior ulnar collateral vessels under the ‘Cubital tunnel’ [5,6,7]. Its roof is formed by the Osborne's ligament or the Cubital retinaculum which is a ligament spanning from the medial epicondyle to the olecranon process, continuous with the fascia connecting the humeral and ulnar heads of the ﬂexor carpi ulnaris (FCU) [figure 1c,d,e]. Alternatively, the roof may be formed by the anconeus epitrochearlis muscle [8]. The floor is formed by the medial collateral ligament (MCL) and elbow joint capsule, while the medial epicondyle and olecranon form the walls on either side [9]. Flexion of the elbow decreases the height, area and sagittal curvature of the tunnel, with maximum nerve compression occurring at 135 degrees of flexion according to a study by James et al. [10] At this level, the ulnar nerve gives off an articular branch to the elbow joint.

Thereafter, the ulnar nerve descends into the forearm, passing between the two heads of the flexor carpi ulnaris muscle (FCU) [11]. The nerve provides motor supply to the FCU and medial half of the flexor digitorum profundus. It continues distally along the ulna, lying deep to the FCU. About 5 cm distal to the medial epicondyle, the ulnar nerve pierces the flexor-pronator aponeurosis which is the fibrous common origin of the flexor and pronator muscles. Another aponeurosis extends between the flexor digitorum superficialis of the ring finger and the humeral head of the flexor carpi ulnaris, known as the ligament of Spinner, which attaches to the medial epicondyle and can cause kinking of the nerve following an anterior transposition.

Near the wrist, the ulnar nerve moves lateral to the FCU, courses medial to the ulnar artery to enter the palm through the Guyon’s canal. The volar carpal ligament forms the roof of the canal while the transverse carpal ligament and the hypothenar muscles form the floor. The medial and lateral walls are formed by pisiform, pisohamate ligament, abductor digiti minimi muscle belly on the ulnar side and the hook of hamate on the radial side [12]. Near the wrist, the motor innervation of the ulnar nerve includes the thenar muscles: the adductor policis, deep head of flexor pollicis brevis (FPB); dorsal and palmar interossei and 3rd and 4th lumbricals, hypothenar muscles: abductor digiti minimi, opponens digiti minimi and flexor digiti minimi. The ulnar nerve gives dorsal and palmar cutaneous branches and a few superficial terminal branches to provide sensation to the ulnar fourth and entire fifth finger and the medial aspect of the forearm. (Figure 1f,g,h)

RADIAL NERVE (Figure 1i-1n)

The radial nerve originates from the ventral rami of C5 – T1 and lies in the posterior cord of the brachial plexus with the axillary artery as its anterior relation. Within the axilla, it has three branches: sensory posterior cutaneous nerve of the arm and motor branch to the long and medial head of the triceps. It passes anterior to the subscapularis, latissimus dorsi and teres major. This is the first possible site of compression. An anomalous muscle, the accessory subscapularis-teres-latissimus is reported in literature to be another cause of compression of the radial nerve at this level [13]. Another potential cause of compression, as described by Spinner [14] is penetration of the nerve directly by the subscapular artery more distally in the axilla, forming a neural loop. It enters the posterior compartment of the arm by travelling through the triangular interval bounded by the teres major superiorly, the long head of the triceps medially and the lateral head of the triceps laterally. The radial nerve then exits the axilla, courses through the lateral head of triceps brachii and winds around the spiral groove accompanied by the profunda brachii artery. Lorem et al. [15] described this as another possible site of entrapment. Familial radial nerve entrapment syndrome [16] also occurs secondary to compression at the lateral head of the triceps. Intermittent compression of the nerve may also occur secondary to genetic defect in Schwann cell myelin metabolism [17]. The radial nerve gives off the following branches in the upper arm - the inferior lateral cutaneous nerve of the arm, posterior cutaneous nerve of the forearm and motor branches to the lateral head of triceps and anconeus. The nerve is in close proximity to the humerus in the spiral groove and is easily identified on ultrasound in its short axis overlying the humerus between the lateral and medial heads of the triceps. (Figure 1j,k,l).

The nerve then courses from the posterior to anterior compartment of the arm by piercing the lateral intermuscular septum which is another possible site of entrapment. Thereafter, it descends in the arm and comes to lie between the brachialis and brachioradialis giving motor supply to both the muscles in addition to the extensor carpi radialis longus (ECRL) and extensor carpi radialis brevis (ECRB). This too is a constant relation which can be identified on ultrasound when scanning the lateral aspect of the lower arm (Figure 1m,n). The nerve courses anterior to the lateral epicondyle into the forearm and divides into superficial and deep branches (posterior interosseous nerve, PIN). The PIN winds around the radial head, lying between two heads of the supinator muscle and then travels on the dorsal surface of the interosseous membrane.

The superficial layer of the supinator muscle forms the arcade of Frohse which is the most common site of entrapment neuropathy causing the radial tunnel syndrome [18]. Two anomalous courses of the PIN have been reported in the literature by Woltman and Learmonth. First is passage of the nerve within the substance of the supinator, and the other involves a branch travelling superficial to the supinator brevis [19]. The origin of the radial tunnel is where the deep branch (lateral) courses over the radio-humeral joint. The tunnel ends at the distal edge of the superficial supinator. Radial tunnel syndrome can be caused by the arcade of Frohse, fibrous fascial bands coursing superficial to the nerve or the vascular leash of Henry [20] which is formed by the recurrent radial vessels. The PIN supplies the abductor pollicis longus, the extensor pollicis brevis, the extensor indicis proprius and the extensor pollicis longus. The superficial branch courses medially, deep to the brachioradialis, lateral to the radial artery into the wrist. It pierces the lateral fascia to enter the anatomical snuffbox and gives sensory innervation to the dorsal surface of three and a half digits on the radial aspect. It also innervates the extensor digitorum, the extensor digiti minimi and the extensor carpi ulnaris muscles.

MEDIAN NERVE [Figure 1o-1q]

C5-T1 roots of the brachial plexus give rise to medial, lateral and posterior cords. The former two cords fuse anterior to the axillary artery to form the median nerve, also known as the ‘eye of the hand’. The nerve descends in the arm lying close to the brachial artery and crosses it anteriorly to lie medially at the elbow. The first site of possible compression is under the Struther’s ligament which is an anomalous bony spur, the supracondylar process, located at the distal humerus, approximately 3-5 cm proximal to the medial epicondyle and 2-20 mm long [21]. The ligament connects this bony spur to the medial epicondyle encasing the median nerve and brachial artery in 13% of the population [22]. Sometime, only the ligament may be present in absence of the bony spur [23,24]. The nerve then enters the cubital fossa, lying lateral to the brachialis muscle and exits the fossa by passing between the two heads of the pronator teres. This is a possible site of compression likely due to anomalous origin of ulnar head, tendinous and short ulnar head or multiple origin slips [25,26]. The bicipital aponeurosis form the roof of the cubital tunnel, partially covering the median nerve. Rare instances of overgrowth or expansion of this ‘lacertus fibrosus’ may entrap the median nerve [27].

It descends in the forearm, lying between the flexor digitorum superficialis (FDS) and flexor digitorum profundus (FDP) and provides innervation to FDS, flexor carpi radialis and palmaris longus. Anomalous aponeurotic arches from FDS may compress the nerve in the forearm [28]. Within the pronator teres, it gives off another branch, the Anterior interosseous nerve (AIN) which supplies the pronator quadratus, lateral half of FDP, flexor pollicis longus (FPL). Compressive neuropathy of AIN is known as Kiloh-Nevin syndrome, which may occur due to trauma, anomalous accessory head of FPL, fibrous arch of FDS, fascial bands of deep head of pronator teres [29,30]. Just proximal to the wrist it gives off the palmar cutaneous branch which gives sensory supply to the lateral aspect of the palm [31].

The median nerve then enters the carpal tunnel, the most common site of entrapment and the most common mononeuropathy [32]. The carpal tunnel is formed by a deep carpal arch which is converted into a tunnel by the flexor retinaculum. The lateral border is formed by the scaphoid and trapezium tubercles while the medial border is formed by the hook of the hamate and pisiform [Figure 1p,q]. The normal CSA of the median nerve at carpal tunnel inlet (level of pisiform) is 9.1 (+/-2.3) mm^2^ and at outlet (level of hamate) is 8.8 (+/-1.8) mm^2^ while the normal CSA of the carpal tunnel is 164 (+/-16.6) mm^2^ at the inlet [33].

Within the tunnel, the median nerve lies anterolateral to the tendons of FDS and FDP, with the FPL being its lateral relation. The flexor carpi radialis does not lie within the tunnel, but is placed under the retinaculum. The median nerve gives off recurrent branches to the thenar muscles and terminates as palmar digital branch providing sensory supply to the palmar surface and fingertips of the lateral three and half digits and motor supply to the lateral two lumbricals.

SCIATIC NERVE [Figure 1r-1u]

The sciatic nerve is derived from the lumbosacral plexus and is the largest nerve of the body. It leaves the pelvis and enters the gluteal region through the greater sciatic foramen and descends in an inferolateral direction [Figure 1s]. It enters the posterior thigh by passing deep to the long head of the biceps femoris and supplies the hamstring muscles and adductor magnus. At the apex of the popliteal fossa, it terminates by bifurcating into the tibial and common fibular nerves. This bifurcation may rarely occur high up in the thigh or sometimes at the plexus itself [34] [Figure 1t,u].

The tibial nerve courses through the popliteal fossa into the posterior compartment of the leg supplying the superficial and deep muscles in the posterior compartment. In the leg, it gives off the medial sural cutaneous nerve which joins with anastomotic rami from the peroneal nerve to form the sural nerve. The tibial nerve descends in the leg, passes behind the medial malleolus and enters the foot by passing through the tarsal tunnel. This osseofibrous tunnel is formed by the medial walls of the distal tibia, talus, sustentaculum tali and calcaneum bodies with the flexor retinaculum forming its roof [35,36,37]. The tibial nerve further branches into the medial plantar nerve, lateral plantar nerve and medial calcaneal nerve, as it tracks infero-posteriorly to the medial malleolus [38].

The second division of the sciatic nerve is the common peroneal nerve. It originates at the level of distal femur and enters the lateral compartment of leg where it winds around neck of fibula, lying deep to the peroneus longus muscle. This is the most common site of common peroneal nerve involvement which may be post traumatic, compressive or due to entrapment [39]. It gives off two sensory nerves, the sural communicating nerve and lateral sural cutaneous nerve. It passes between the attachments of peroneus longus and then divides into superficial and deep branches. The superficial branch innervates the lateral compartment muscles while the deep branch supplies the muscles of the anterior compartment. The superficial peroneal nerve emerges through the peroneal musculature about halfway down the anterolateral aspect of the lower leg. This exit point is located at the level of a defect in the crural fascia, typically about 12 cm above the ankle joint [40]. Beyond this, the nerve is sensory and supplies dorsum of the foot, except the first web space. This site of exit from the fascia is a potential site of entrapment of the nerve.

The deep peroneal nerve descends, along with the anterior tibial artery, just anterior to the interosseous membrane [40]. It courses anterior to the ankle, divides into medial and lateral branches of which the medial carries sensations from the first web space while the lateral supplies the extensors of the foot.

REFERENCES

1. Zaidman CM, Seelig MJ, Baker JC, Mackinnon SE, Pestronk A. Detection of peripheral nerve pathology: comparison of ultrasound and MRI. Neurology. 2013;80(18):1634-1640. doi:10.1212/WNL.0b013e3182904f3f.
2. Kenneth R. Maravilla and Brian C. Bowen. Imaging of the Peripheral Nervous System: Evaluation of Peripheral Neuropathy and Plexopathy, AJNR: 19, June 1998
3. Neil G, Simon, Jason T, Cynthia T, Chin and Michel kliot. Peripheral nerve imaging, Handbook of Clinical Neurology, Vol. 136 (3rd series) Neuroimaging, Part II. J.C. Masdeu and R.G. Gonzalez, Editors © 2016 Elsevier..
4. Edie eneditoCaetanoa, João JoséSabongi, NetobLuiz Angelo, VieiraaMaurício, FerreiraCaetano. Revista; The arcade of Struthers: an anatomical study and clinical implications. Brasileira de Ortopedia (English Edition) Volume 52, Issue 3, May–June 2017, Pages 331-336
5. Andrews, K., Rowland, A., Pranjal, A., & Ebraheim, N. Cubital tunnel syndrome: Anatomy, clinical presentation, and management. Journal of Orthopaedics, 2018, 15(3), 832–836. doi:10.1016/j.jor.2018.08.010
6. Granger A, Sardi JP, Iwanaga J, et al. Osborne's ligament: a review of its history, anatomy, and surgical importance. Cureus. 2017;9(3):e1080.
7. Kazuteru Doi, PRINCIPLES OF RECONSTRUCTIVE SURGERY, in Flaps and Reconstructive Surgery, 2009, section 1, pg 71-79. <https://doi.org/10.1016/B978-0-7216-0519-7.00008-3>
8. Folberg CR, Weiss AP, Akelman E. Cubital tunnel syndrome. Part I: presentation and diagnosis. Orthop Rev. 1994;23(2):136–144.
9. Huang JH, Samadani U, Zager EL. Ulnar nerve entrapment neuropathy at the elbow: simple decompression. Neurosurgery. 2004;55(5):1150–1153.
10. James J, Sutton LG, Werner FW, Basu N, Allison MA, Palmer AK. Morphology of the cubital tunnel: an anatomical and biomechanical study with implications for treatment of ulnar nerve compression. J Hand Surg Am. 2011;36(12):1988–1995.
11. Posner MA.Compressive neuropathies of the ulnar nerve at the elbow and wrist.Instr Course Lect. 2000;49:305–317.
12. Shen, L., Masih, S., Patel, D. B., & Matcuk, G. R. (2016). MR anatomy and pathology of the ulnar nerve involving the cubital tunnel and Guyon’s canal. Clinical Imaging, 40(2), 263–274. doi:10.1016/j.clinimag.2015.11.008
13. Kameda Y (1976) An anomalous muscle (accessory subscapularisteres- latissimus muscle) in the axilla penetrating the brachial plexus in man. Acta Anat (Basel) 96:513–533
14. SpinnerM(1980) Management of nerve compression lesions of the upper extremity. In: Omer GE Jr, Spinner M (eds) Management of Peripheral Nerve Problems. WB Saunders, Philadelphia, pp 569– 587
15. Lotem M, Fried A, Levy M, Solzi P, Najenson T, Nathan H (1971) Radial palsy following muscular effort: a nerve compression syndrome possibly related to a fibrous arch of the lateral head of the triceps. J Bone Joint Surg Br 53:500–506
16. Lubahn JD, Lister GD (1983) Familial radial nerve entrapment syndrome: a case report and literature review. J Hand Surg Am 8: 297–299
17. Mayer RF, Garcia-Mullin R (1968) Hereditary neuropathy manifested by pressure palsies: Schwann cell disorder? TransAmNeurol Assoc 93:238–240
18. Filler AG, Kliot M, Howe FA, et al. Application of magnetic resonance neurography in the evaluation of patients with peripheral nerve pathology. J Neurosurg 1996; 85:299–309
19. Dailey AT, Tsuruda JS, Filler AG, Maravilla KR, Goodkin R, Kliot M. Magnetic resonance neurography of peripheral nerve degeneration and regeneration. Lancet 1997; 350:1221–1222.
20. Heinemeyer O, Reimers CD. Ultrasound of radial, ulnar, median, and sciatic nerves in healthy subjects and patients with hereditary motor and sensory neuropathies. Ultrasound Med Biol, 1999; 25:481–485.
21. Varlam H, St Antohe D, Chistol RO. [Supracondylar process and supratrochlearforamen of the humerus: a case report and a review of the literature]. Morphologie. 2005 Sep. 89(286):121-5.
22. Siqueira MG, Martins RS. The controversial arcade of Struthers. Surg Neurol. 2005. 64 Suppl 1:S1:17-20; discussion S1:20-1. [Medline].
23. Suranyi L. Median nerve compression by Struthers ligament. J Neurol Neurosurg Psychiatry. 1983 Nov. 46(11):1047-9. [Medline].
24. Gessini L, Jandolo B, Pietrangeli A. Entrapment neuropathies of the median nerve at and above the elbow. Surg Neurol. 1983 Feb. 19(2):112-6.
25. Wertsch JJ, Melvin J. Median nerve anatomy and entrapment syndromes: a review. Arch Phys Med Rehabil. 1982 Dec. 63(12):623-7. [Medline].
26. Bilecenoglu B, Uz A, Karalezli N. Possible anatomic structures causing entrapment neuropathies of the median nerve: an anatomic study. Acta Orthop Belg. 2005 Apr. 71(2):169-76.
27. Swiggett R, Ruby LK. Median nerve compression neuropathy by the lacertus fibrosus: report of three cases. J Hand Surg Am. 1986 Sep. 11 (5):700-3.
28. Dellon AL, Mackinnon SE. Musculoaponeurotic variations along the course of the median nerve in the proximal forearm. J Hand Surg Br. 1987 Oct. 12 (3):359-63
29. Degreef I, De Smet L. Anterior interosseous nerve paralysis due to Gantzer's muscle. Acta Orthop Belg. 2004 Oct. 70(5):482-4. [Medline].
30. al-Qattan MM. Gantzer's muscle. An anatomical study of the accessory head of the flexor pollicis longus muscle. J Hand Surg Br. 1996 Apr. 21 (2):269-70
31. Kermarrec E, Demondion X, Khalil C, Le Thuc V, Boutry N, Cotten A. Ultrasound and magnetic resonance imaging of the peripheral nerves: current techniques, promising directions, and open issues. Semin Musculoskelet Radiol 2010; 14:463–472.
32. Pätiälä H, Rokkanen P, Kruuna O, Taponen E, Toivola M, Häkkinen V. Carpal tunnel syndrome. Anatomical and clinical investigation. Arch Orthop Trauma Surg. 1985. 104 (2):69-73. [Medline].
33. Quantitative MRI of the wrist and nerve conduction studies in patients with idiopathic carpal tunnel syndrome, S Uchiyama, T Itsubo, T Yasutomi, H Nakagawa, M Kamimura, H Kato, J Neurol Neurosurg Psychiatry 2005;76:1103–1108. doi: 10.1136/jnnp.2004.051060
34. Khan AA, Asari MA, Pasha MA. Folia Morphol (Warsz), The sciatic nerve in human cadavers - high division or low formation?. 2016;75(3):306-310. doi: 10.5603/FM.a2015.0130. Epub 2015 Dec 29. PMID: 26711654
35. Ahmad, M., et al., Tarsal tunnel syndrome: a literature review. Foot and Ankle Surgery, 2012. 18(3): p. 149-152
36. Machiels, F., et al., Tarsal tunnel syndrome: ultrasonographic and MRI features. JBR-BTR: organe de la Societe royale belge de radiologie (SRBR)= orgaan van de Koninklijke Belgische Vereniging voor Radiologie (KBVR), 1999. 82(2): p. 49-50.583 21.
37. McMinn RM., H.R., Logan BM. , Foot & Ankle Anatomy, 2nd edition. . 1996, London: Times Mirror International Publishers Limited.
38. Dellon, A.L. and S.E. Mackinnon, Tibial nerve branching in the tarsal tunnel. Archives of neurology, 1984. 41(6): p. 645-646
39. Sunderland S, Ray LJ. The intraneural topography of the sciatic nerve and its popliteal divisions in man. Brain 1948;71(Pt. 3):242–73.
40. Sarrafian S, Kelikian A. Nerves. In: Kelikian A, Sarrafian S, eds. Sarrafian’s anatomy of the foot and ankle. 3rd ed. Philadelphia, Pa: Wolters Kluwer–Lippincott Williams & Wilkins, 2011; 381–427.

FIGURE LEGENDS:
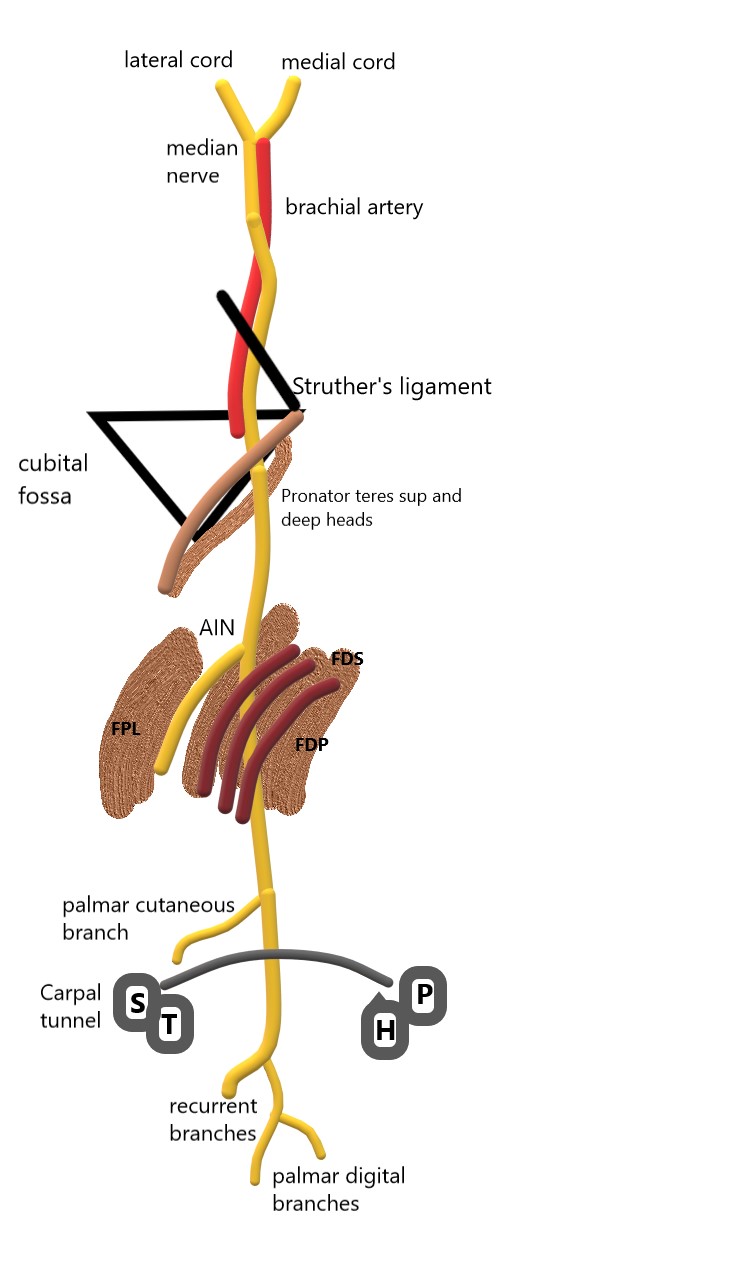


Figure 1b: Diagrammatic representation of the course of ulnar nerve and its anatomical relations with possible sites of compression.


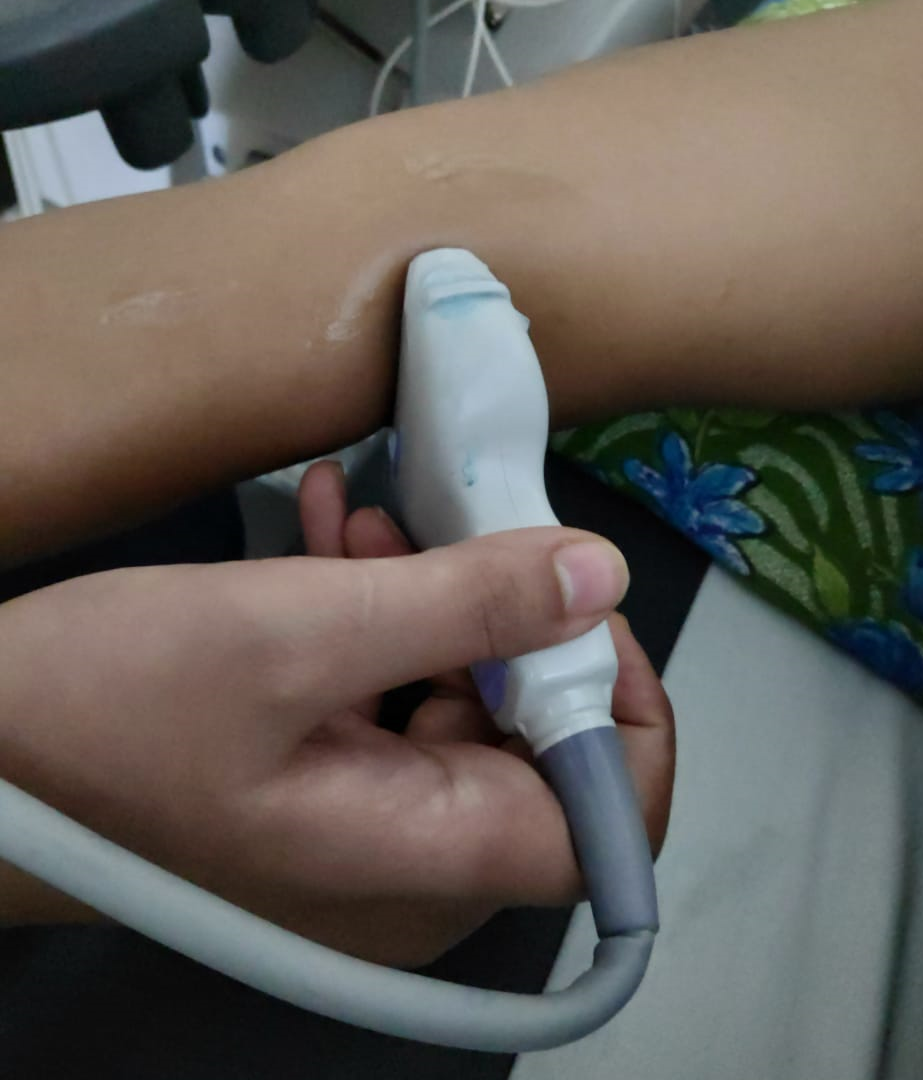


Figure 1c: Probe position for localisation of the ulnar nerve at the elbow.


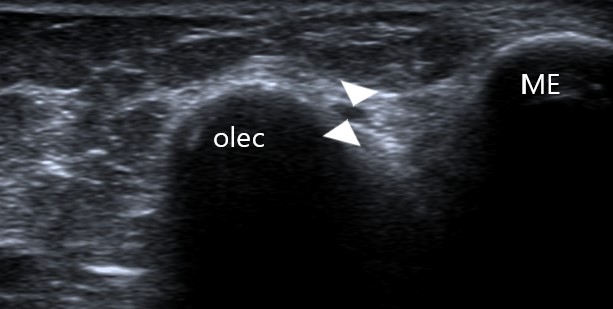
Figure 1d: Ulnar nerve (arrowhead) is localised in its short axis within the cubital tunnel between the medial epicondyle (ME) and olecranon process (olec).


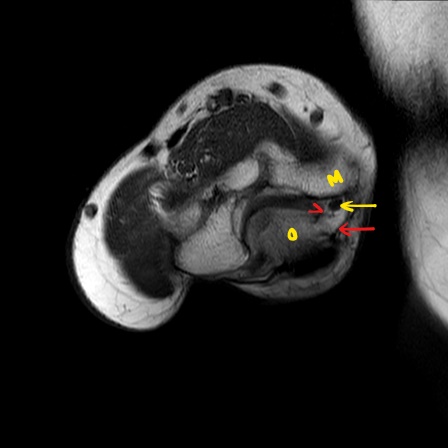
Figure 1e: MR image showing the normal ulnar nerve (yellow arrow) in the cubital tunnel: Medial epicondyle (M) and olecranon process (O) forming the medial and lateral walls, Osborne’s ligament (red arrow) forming the roof and the floor being formed by the joint capsule of elbow and the medial collateral ligament (red arrowhead).


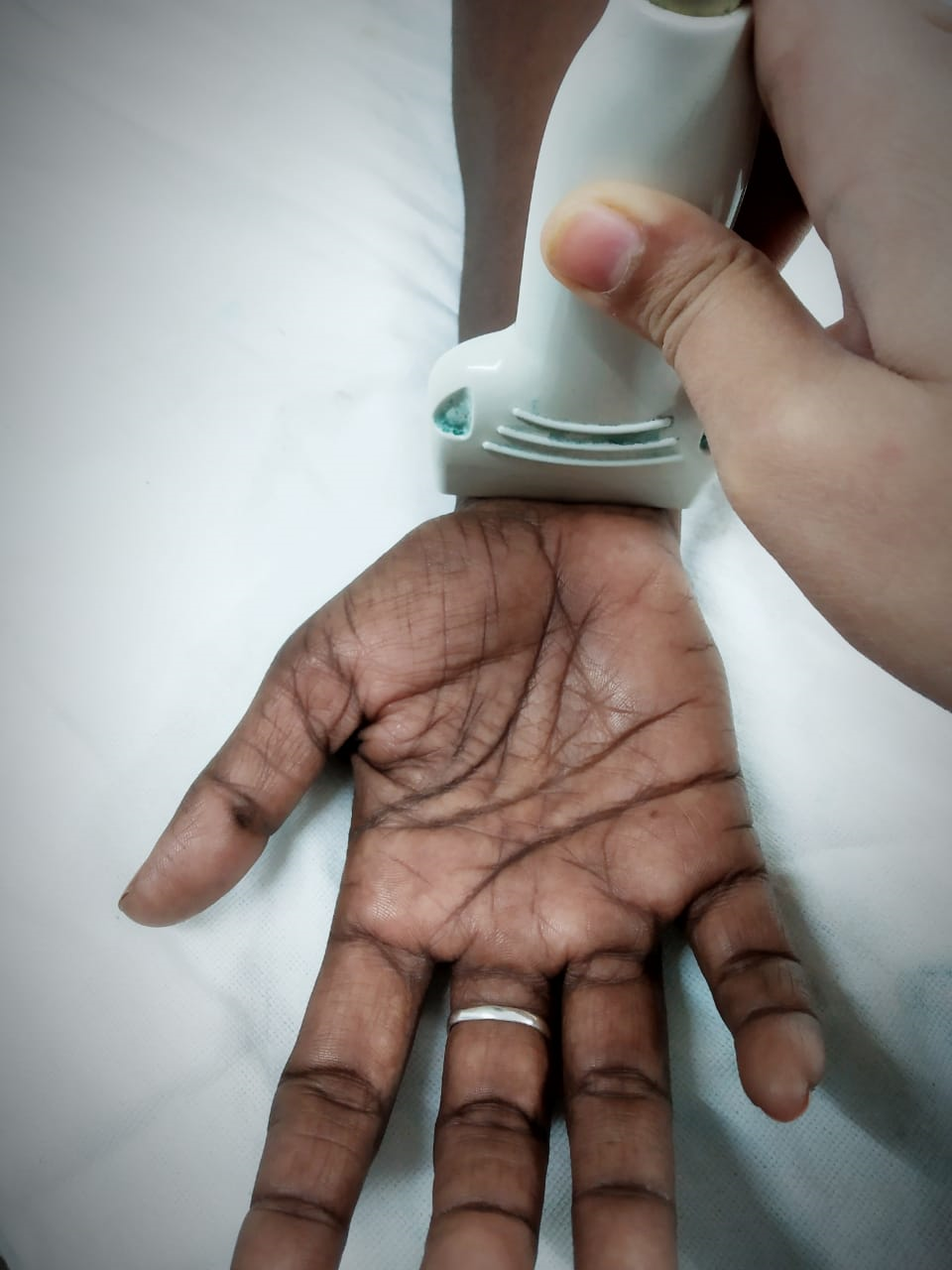
Figure 1f: Probe position for localisation of the ulnar nerve at the wrist.


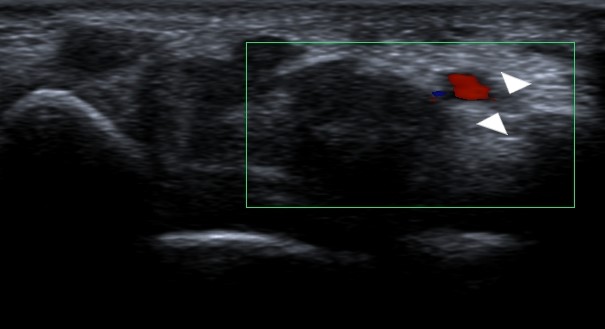
Figure 1g: The ulnar nerve (arrowhead) is localised in the short axis in the Guyon’s canal between the pisiform and hook of hamate, lying adjacent to the ulnar artery (colour doppler signal).


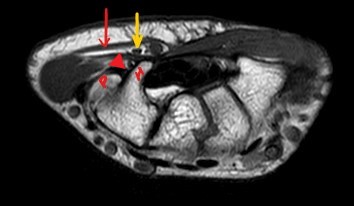


Figure 1h: MR image showing the normal ulnar nerve (yellow arrow) in the Guyon’s canal: pisiform (P) and the hook of hamate (H) form the radial and ulnar boundaries. The roof is formed by the volar carpal ligament (red arrow) while the hypothenar muscles and transverse carpal ligament (red arrowhead) form the floor.


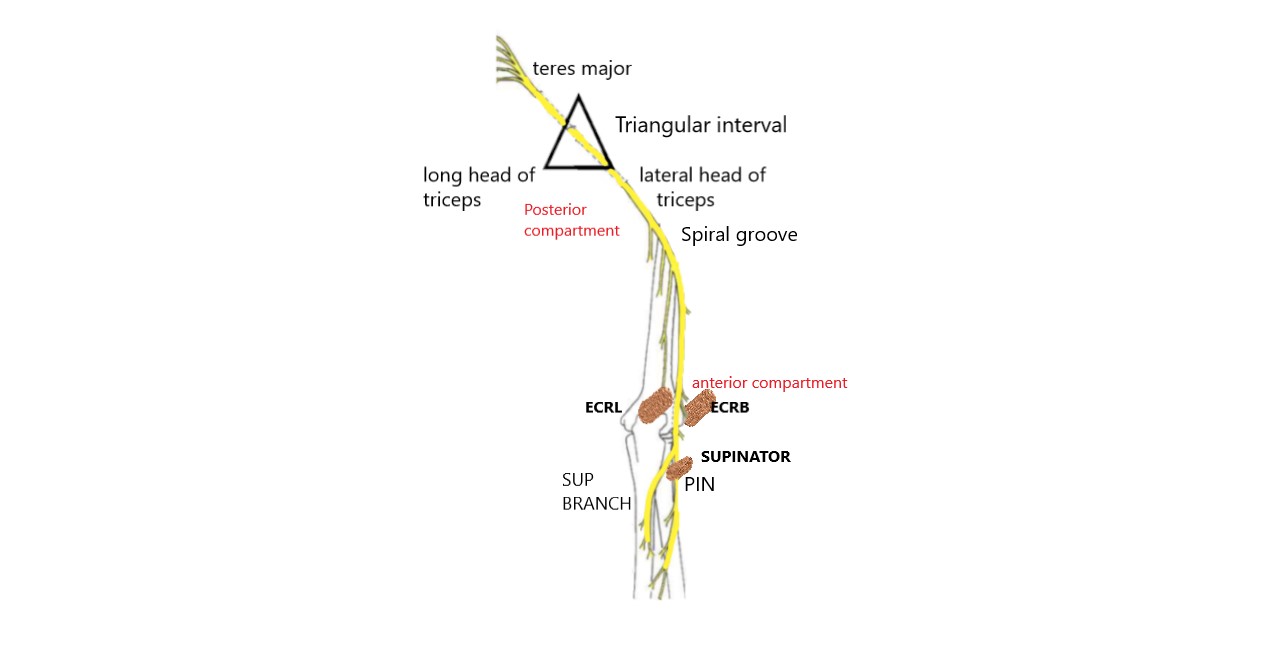
Figure 1i: Diagrammatic representation of the course of radial nerve and its anatomical relations with possible sites of compression.


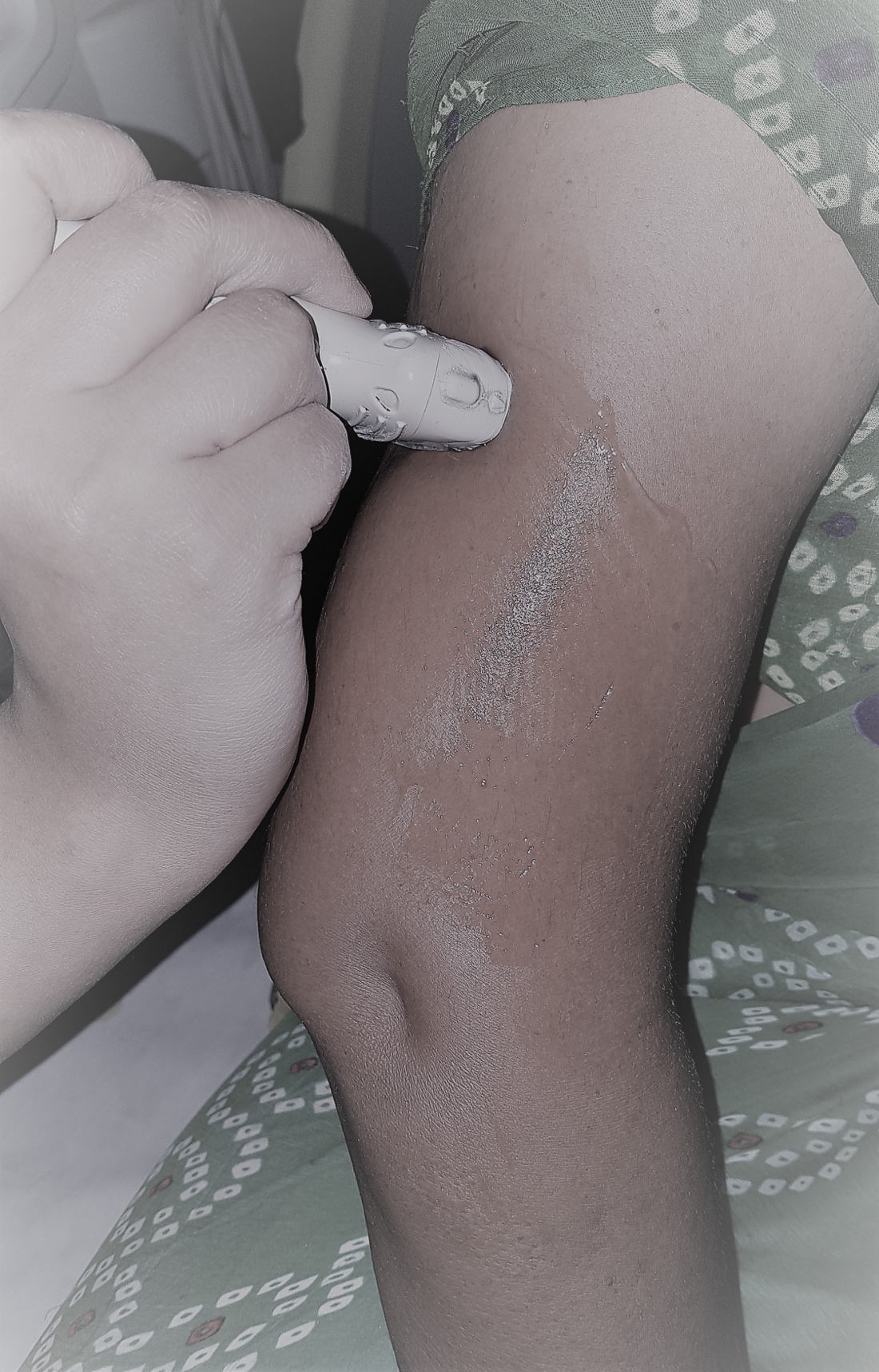


Figure 1j: Probe position for localising the radial nerve in spiral groove. The patient’s arm is kept extended, internally rotated and posterolateral aspect of the arm is scanned.


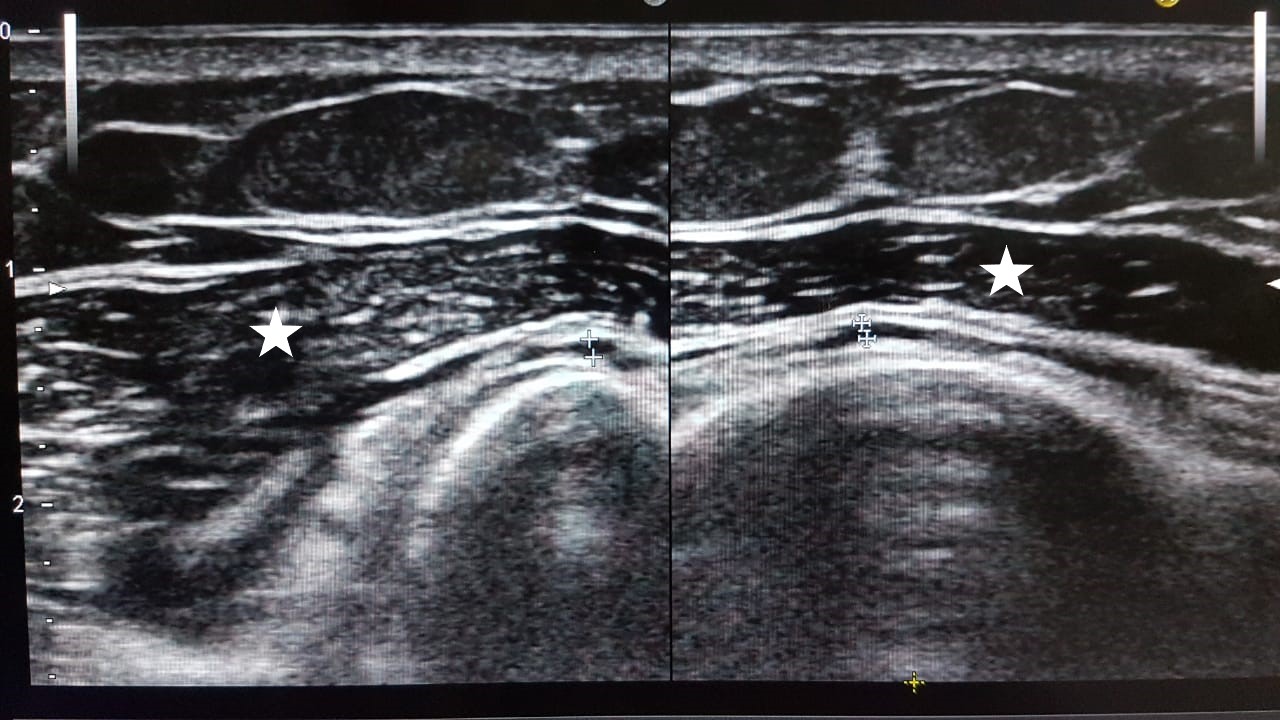
Figure 1k: Cross heads denote the normal honeycomb appearance of radial nerve in the spiral groove between lateral and medial heads of triceps (asterisk)


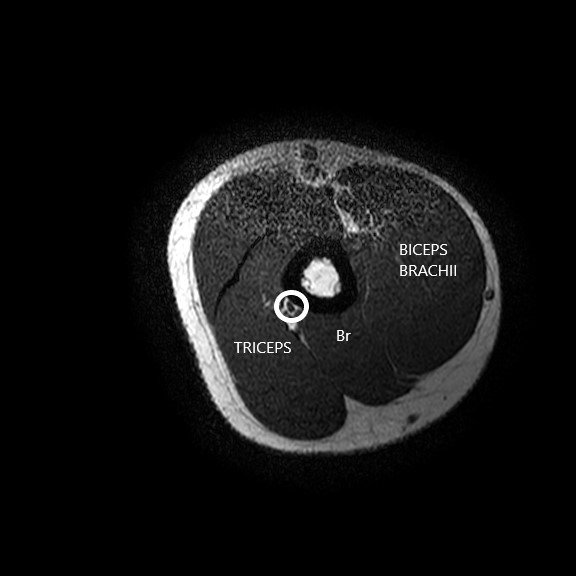
Figure 1l: Axial T1 weighted MRI at the level of the spiral groove shows the radial nerve (encircled). Br: brachialis.


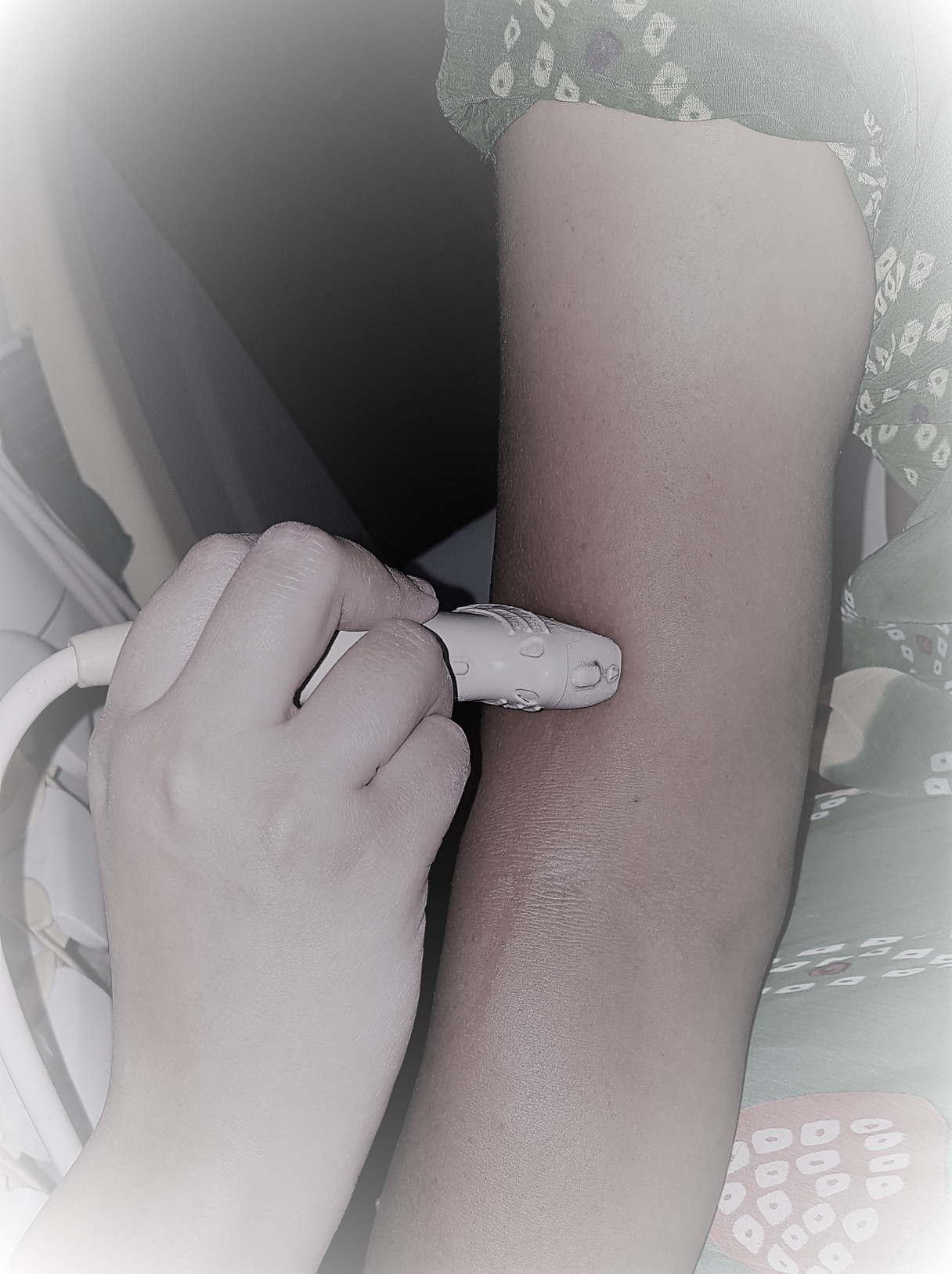
Figure 1m: Probe position for localising the nerve between brachialis and brachioradialis. The lateral part of arm is to be scanned with arm flexed at elbow and internally rotated.


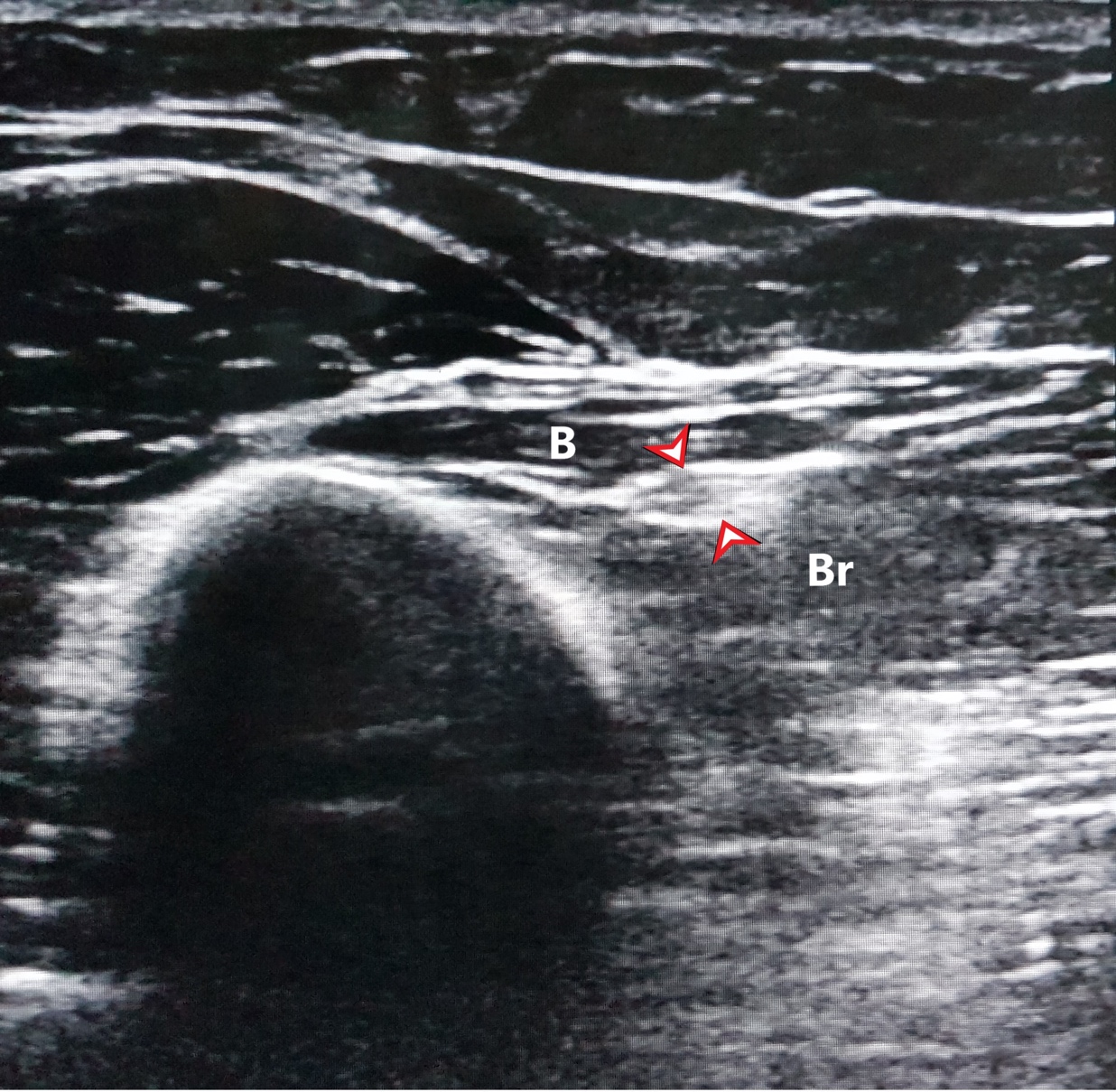
Figure 1n: Arrowheads denote the normal honeycomb appearance of radial nerve in distal arm lying between brachialis (B) and brachioradialis (Br).


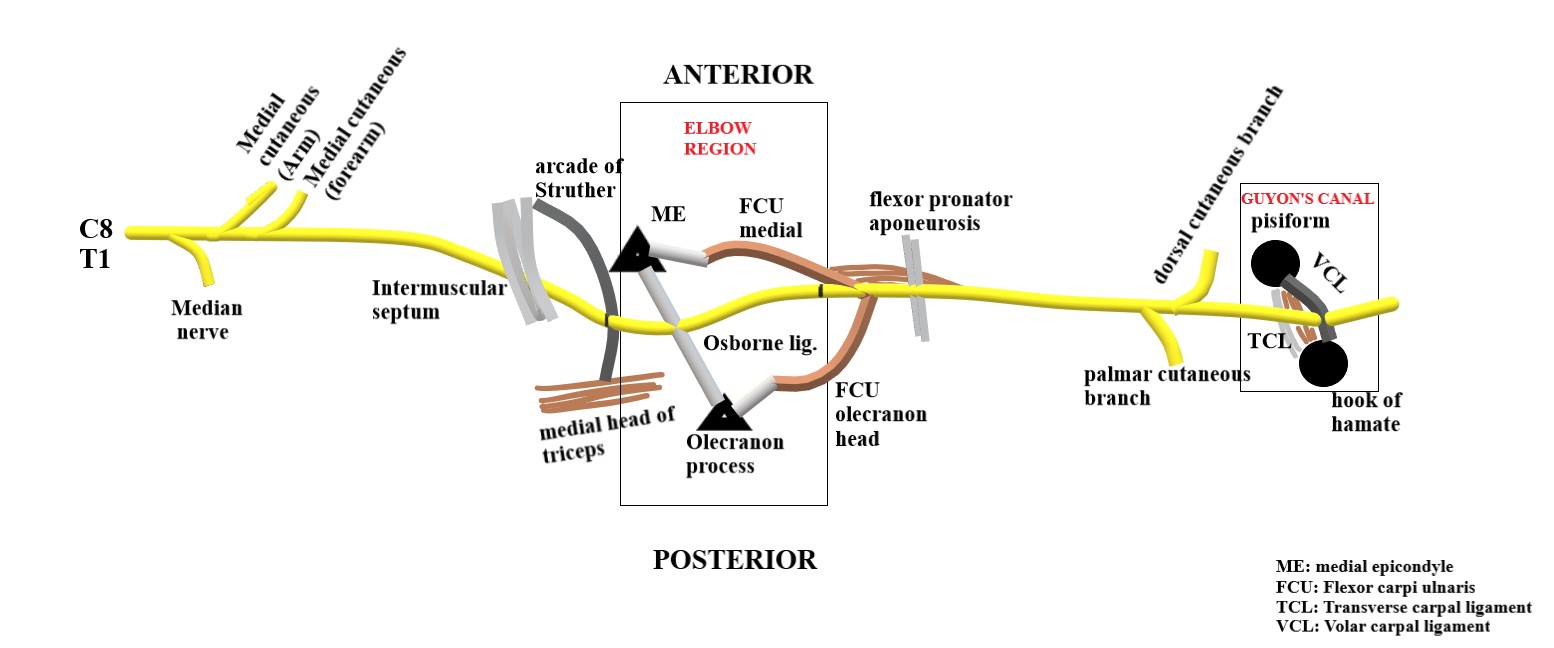
Figure 1o: Diagrammatic representation of the course of median nerve and its anatomical relations with possible sites of compression.


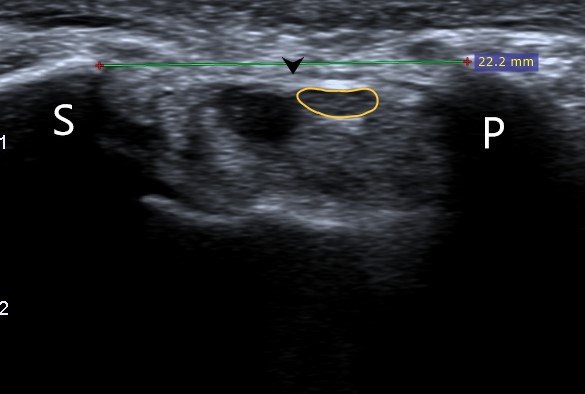
Figure 1p: Transverse HRUS scan at the level of wrist showing the median nerve (yellow outline) lying below the flexor retinaculum (arrowhead) at the carpal tunnel inlet (between scaphoid (S) and pisiform (P)). There is no bowing of the flexor retinaculum in this normal subject as evident by the flexor retinaculum lying below the horizontal line joining the S and P.


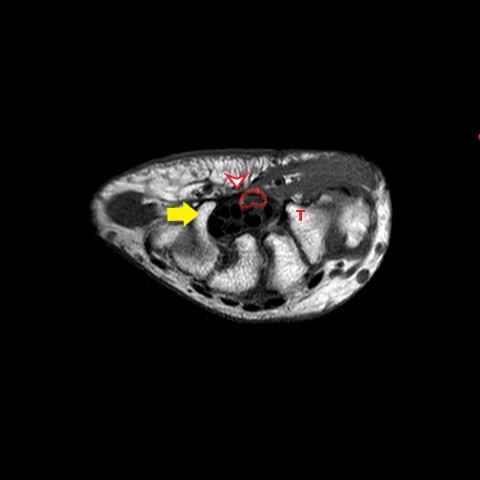


Figure 1q: Axial T1 weighted image at the level of the carpal tunnel outlet (between the hook of hamate (yellow arrow) and trapezium (T) showing the median nerve (red outline) and the flexor retinaculum (arrowhead).


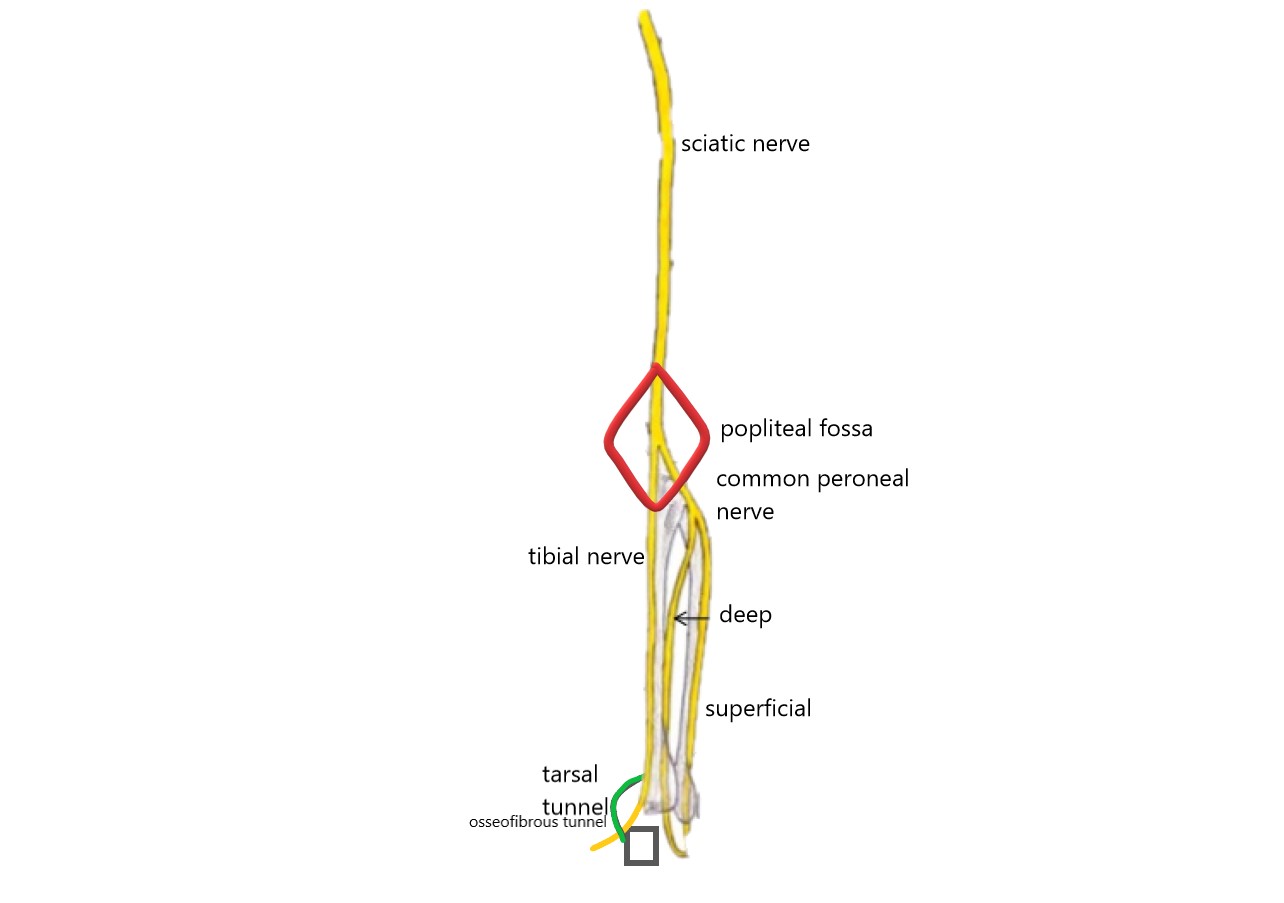
Figure 1r: Diagrammatic representation of the course of sciatic nerve, its branches and its anatomical relations with possible sites of compression.


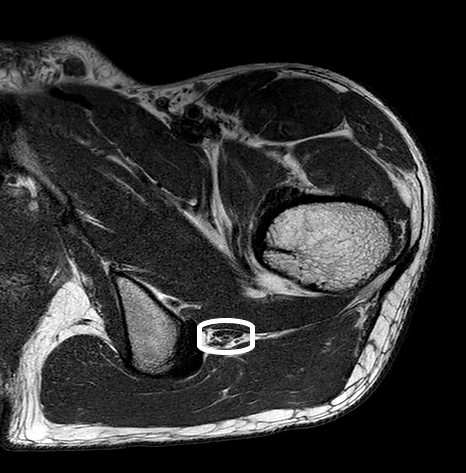


Figure 1s: The sciatic nerve (encircled) with the characteristic honeycomb architecture is seen on this T1 weighted axial image, just after exiting the sciatic foramen.


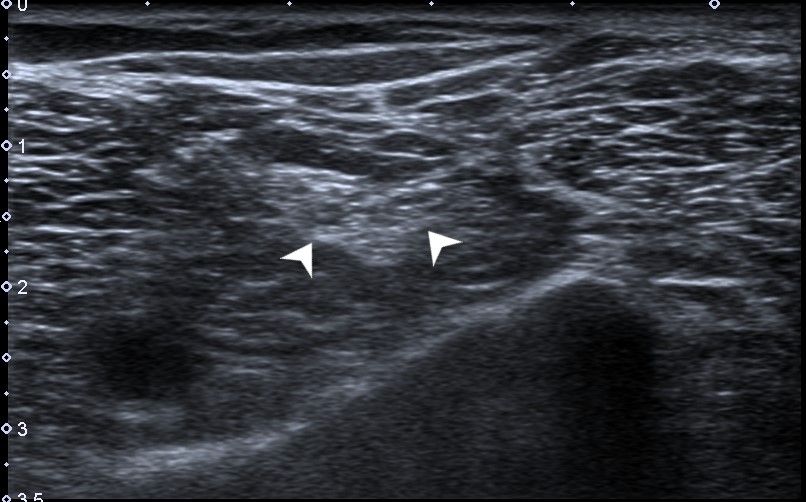
Figure 1t: Transverse HRUS scan just above the popliteal fossa shows the division of the sciatic nerve into the tibial and common peroneal nerve (arrowheads).


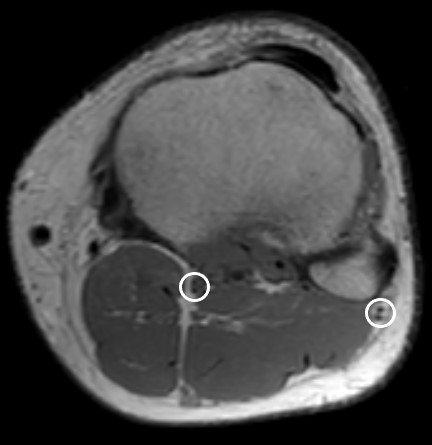


Figure 1u: T1 weighted axial image shows the tibial and common peroneal nerves (CPN) (encircled) in the proximal leg. The CPN can be seen lying in close proximity to the fibular neck.
